# Fluorogenic Peptide Sensor Array Derived from Angiotensin-Converting Enzyme 2 Classifies Severe Acute Respiratory Syndrome Coronavirus 2 Variants of Concern

**DOI:** 10.1021/jacs.4c06172

**Published:** 2024-07-19

**Authors:** Wei-Tao Dou, Pei-Hong Tong, Man Xing, Jiao-Jiao Liu, Xi-Le Hu, Tony D. James, Dong-Ming Zhou, Xiao-Peng He

**Affiliations:** † Key Laboratory for Advanced Materials and Joint International Research Laboratory of Precision Chemistry and Molecular Engineering, Feringa Nobel Prize Scientist Joint Research Center, Frontiers Center for Materiobiology and Dynamic Chemistry, School of Chemistry and Molecular Engineering, East China University of Science and Technology, 130 Meilong Rd., Shanghai 200237, China; ‡ The International Cooperation Laboratory on Signal Transduction, National Center for Liver Cancer, Eastern Hepatobiliary Surgery Hospital, Shanghai 200438, China; § Vaccine and Immunity Research Center, Shanghai Public Health Clinical Center, Fudan University, Shanghai 201508, China; ∥ Department of Pathogen Biology, School of Basic Medical Sciences, Tianjin Medical University, Tianjin 300070, China; ⊥ Department of Chemistry, University of Bath, Bath BA2 7AY, U.K.; # School of Chemistry and Chemical Engineering, Henan Normal University, Xinxiang 453007, China

## Abstract

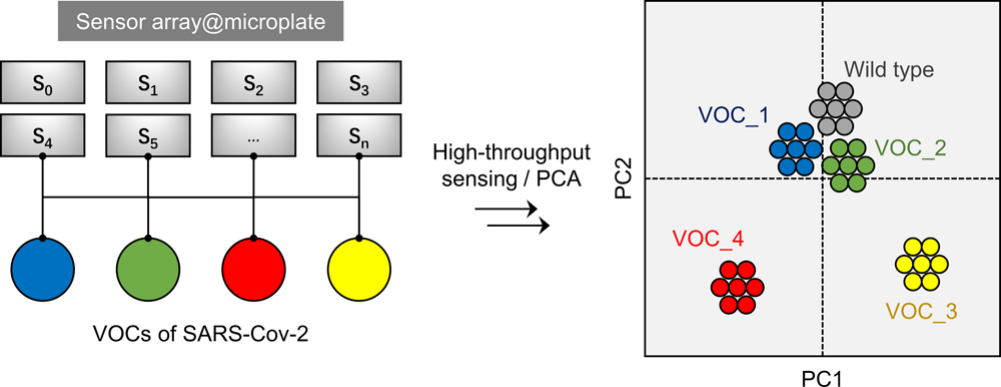

The devastating COVID-19 pandemic caused by severe acute respiratory syndrome coronavirus 2 (SARS-CoV-2) has made society acutely aware of the urgency in developing effective techniques to timely monitor the outbreak of previously unknown viral species as well as their mutants, which could be even more lethal and/or contagious. Here, we report a fluorogenic sensor array consisting of peptides truncated from the binding domain of human angiotensin-converting enzyme 2 (hACE2) for SARS-CoV-2. A set of five fluorescently tagged peptides were used to construct the senor array in the presence of different low-dimensional quenching materials. When orthogonally incubated with the wild-type SARS-CoV-2 and its variants of concern (VOCs), the fluorescence of each peptide probe was specifically recovered, and the different recovery rates provide a “fingerprint” characteristic of each viral strain. This, in turn, allows them to be differentiated from each other using principal component analysis. Interestingly, the classification result from our sensor array agrees well with the evolutionary relationship similarity of the VOCs. This study offers insight into the development of effective sensing tools for highly contagious viruses and their mutants based on rationally truncating peptide ligands from human receptors.

## Introduction

The COVID-19 pandemic, caused by severe acute respiratory syndrome coronavirus 2 (SARS-CoV-2), has underscored the urgent need for a comprehensive understanding of the structure, function, and evolution of the virus. The virological background of COVID-19 is grounded in the intricate molecular and cellular interactions between the virus and human hosts. SARS-CoV-2 targets the human respiratory system, exploiting the angiotensin-converting enzyme 2 (ACE2) receptor to facilitate entry into host cells.^1,2^ This interaction triggers a cascade of biological events, which finally result in characteristic COVID-19 symptoms ranging from mild respiratory distress to severe pneumonia and acute respiratory distress syndrome.^3,4^ Thus, the systematic study of SARS-CoV-2 virology is crucial for developing effective diagnostic and therapeutic strategies.

One of the most pressing challenges in combating viral pandemics is the emergence of variants of concern (VOCs), which exhibit distinct genetic signatures and potentially altered phenotypic characteristics.^5,6^ Timely surveillance and phenotyping of VOCs are essential to effectively adjust public health measures and treatment strategies. Rapid and accurate detection of SARS-CoV-2 and its variants is fundamental to controlling virus spread. Currently, the gold standard for SARS-CoV-2 detection is the use of polymerase chain reaction (PCR), which amplifies viral genetic material for quantitative analysis.^7^ While PCR is highly sensitive and specific, it requires sophisticated laboratory equipment to be used, the analyst to be well-trained, and requires long turnaround times. Antigen tests, which detect representative viral proteins, offer a more convenient alternative to PCR.^8^ However, they are of low sensitivity, particularly for asymptomatic individuals. Additionally, both PCR and antigen tests focus on detecting the presence of a target viral species without providing any detailed information on its genetic features. Given the rapidly evolving nature of SARS-CoV-2, there is growing awareness that effectively phenotyping VOCs may facilitate our understanding of viral evolution thus providing guidance for therapy and vaccination.

Advanced techniques, such as next-generation sequencing (NGS), are increasingly being employed to completely analyze the genome of viral species. NGS allows researchers to identify genetic variants with high resolution, enabling the surveillance of VOCs and their prevalence in different populations. Integrating the acquired genomic data with clinical and epidemiological data may help improve our understanding of how a virus evolves and interacts with human hosts. However, techniques that directly measure the structural or phenotypical traits of viruses are relatively rare. To meet this need, sensing technologies based on electrochemistry,^9,10^ colorimetry,^11^ fluorescence,^12^ chemiluminescence,^13^ and surface plasma resonance^14^ have been developed. While these techniques are simpler in sample processing (e.g., lysis-free and nucleic acid isolation-free) and lower in cost, they still fail to meet the need for classification of viral mutants in their intact form.

**Figure 1:**
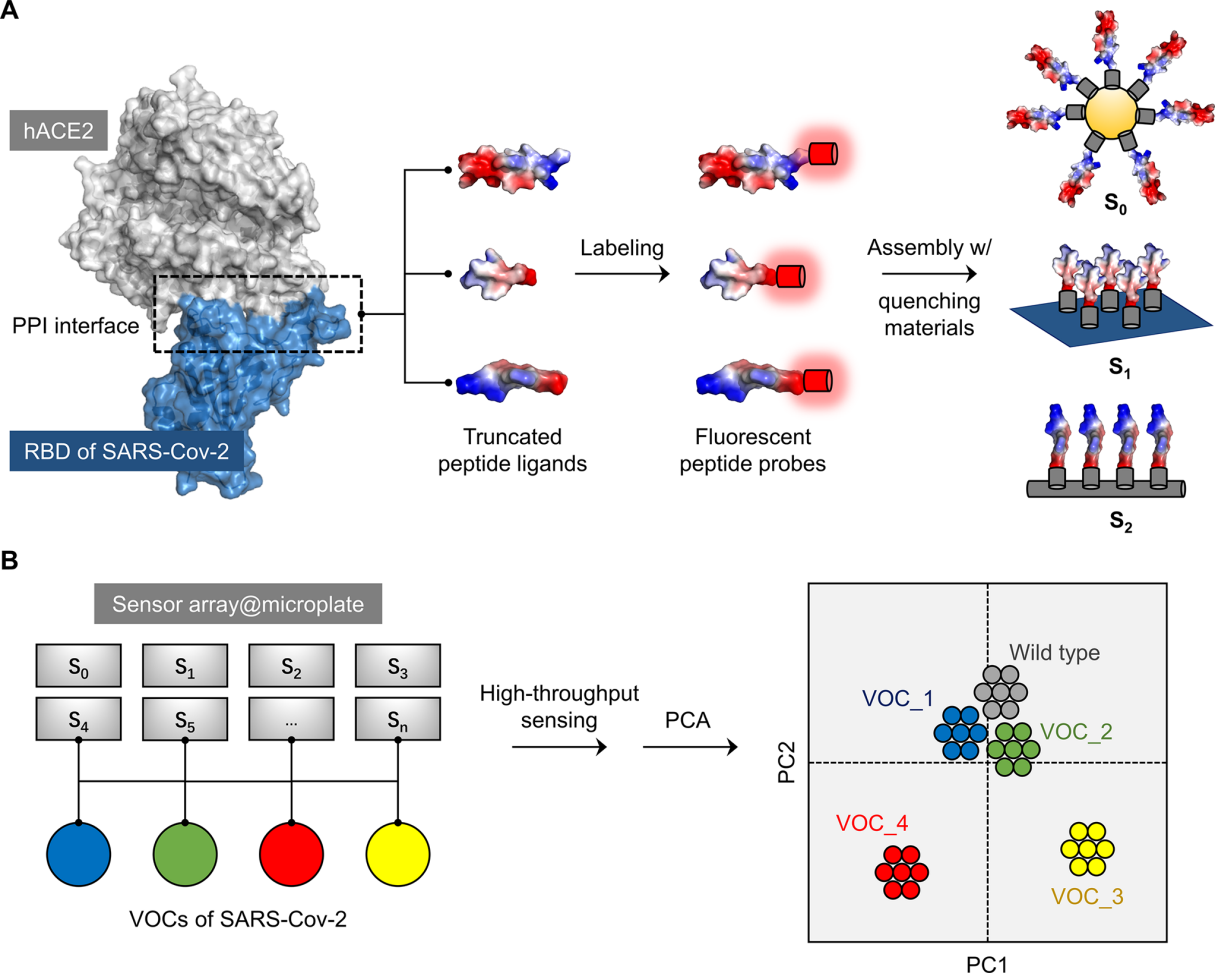
Design and construction of the peptide-based sensor array. (A) Schematic illustration of peptides truncated from the binding domain of hACE2 for SARS-CoV-2 RBD (PDB code of the complex we referred to 6M0J), and the construction of the peptide-based fluorogenic sensor array based on fluorescently tagged hACE2 peptide probes and a variety of quenching materials; PPI means protein–protein interaction. (B) Application of the sensor array for the classification of VOCs of SARS-CoV-2 in the presence of a wild-type (WT) strain. We note that our experimental results suggest that the VOCs with a more distant evolutionary profile to that of WT could be better differentiated.

Here, we developed a fluorogenic sensor array for the high-throughput classification of the World Health Organization (WHO)-announced VOCs of SARS-CoV-2. On the basis of the sensor array concept coined by Anslyn,^15,16^ we sought the exploitation of a set of binding agents that generate moderate interactions with the receptor-binding domain (RBD) of the spike protein (S-protein), the main functional viral protein that mediates entry of SARS-CoV-2 into host cells. We resorted to the interaction interface between S-protein and its human receptor, ACE2,^17^ as we hypothesized that the truncation of key binding peptides from human ACE2 (hACE2) would generate the desired ligands for SARS-CoV-2 with moderate binding affinity. Subsequently, when tagged with a fluorescent dye and mixed with different quenching materials, a fluorogenic sensor array was established (Figure 1A). Indeed, the experimental results detailed below confirm the robustness of the sensor array for accurately classifying VOCs of SARS-CoV-2 with the results agreeing well with the evolutionary similarity of the viral mutants (Figure 1B).

## Results and Discussion

### Design and Preparation of The Fluorescent Peptide Probes

**Figure 2:**
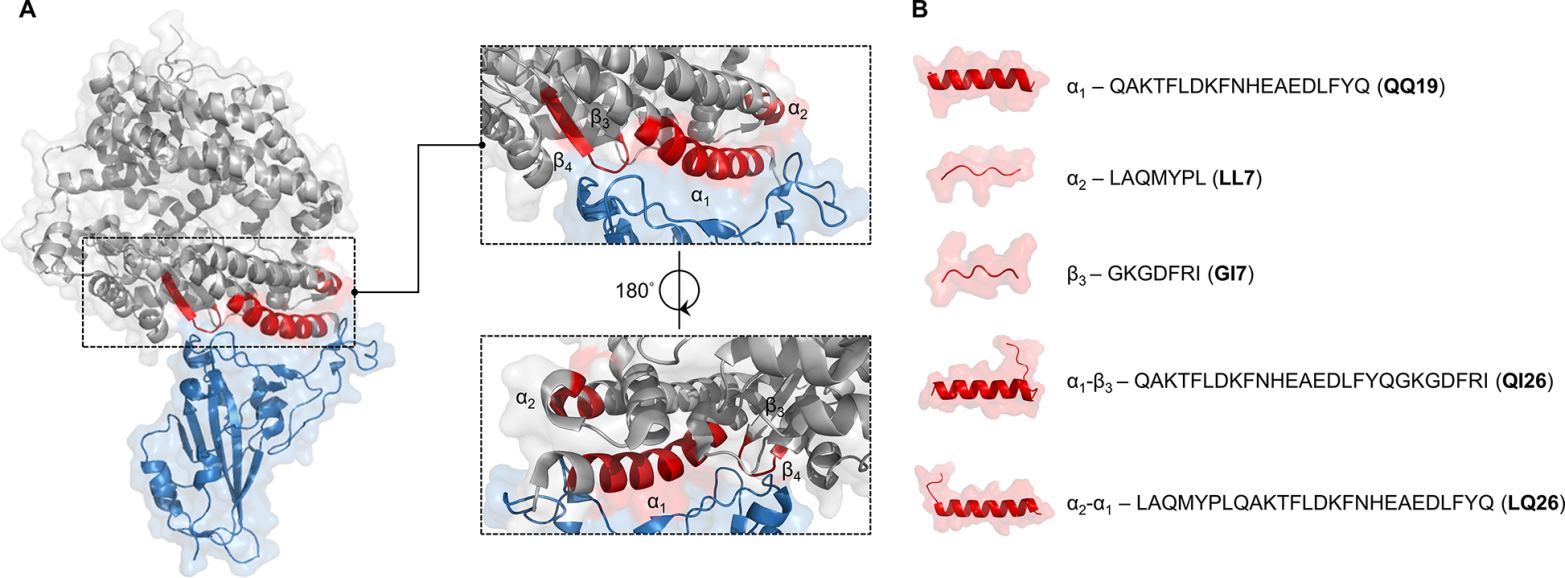
Truncated peptide ligands from hACE2. (A) Structure of the SARS-CoV-2 RBD–hACE2 complex (PDB code: 6M0J), this study refers to in the enlarged view; the blue, gray and red parts represent SARS-CoV-2 RBD, hACE2, and the key peptides truncated from hACE2, respectively. (B) AlphaFold2-simulated secondary structure and amino-acid sequence of the truncated peptide ligands.

hACE2 is the most common endogenous receptor for SARS-CoV-2.^2^ The virus-host cell recognition is initiated by the binding between the RBD of the viral S-protein and the protease domain (PD) of hACE2.^18^ From a crystal structure of hACE2 and RBD derived from SARS-CoV-2 (PDB code: 6M0J),^19^ we noticed that the α_1_-helix, a minor part of the α_2_-helix, and a major part of β_3_ and β_4_-sheet from PD of ACE2 contributed significantly to the protein–protein interactions (Figure 2A). Thus, we sought to truncate these key peptides from hACE2 to construct a sensor array for SARS-CoV-2 variants. These shortened peptides can generate weak-to-moderate interactions with the RBD of the virus, and mutations in the RBD sequence would further change their receptor binding affinity, thus allowing for a diverse set of host–guest binding profiles to be generated. After being processed by multivariate statistic methods such as principal component analysis (PCA), we envision that the different RBDs from VOCs of SARS-CoV-2 could be differentiated.

Based on the hypothesis described above, we prepared a total of five peptide ligands for RBDs. A continuous 19-mer (amino-acid residues 24–42) truncated from the α_1_-helix (**QQ19**), a heptamer (residues 79–85) from α_2_-helix (**LL7**), a heptamer (residues 352–358) from the β_3_ and β_4_-sheet (**GI7**), as well as two spliced peptide hybrids, namely, a 26-mer by splicing the α_1_-19-mer with the β_3_ and β_4_-heptamer (**QI26**), and another 26-mer by splicing the α_1_-19-mer with the α_2_-heptamer (**LQ26**), were synthesized by the solid-phase method (Figure 2B). The structure and purity of the individual peptides were confirmed by mass spectroscopy and high-performance liquid chromatography, respectively (see the Supporting Information). To acquire a detectable signal, the five peptide ligands were tagged with a fluorescent dye (Scheme S1), 5-carboxytetramethylrhodamine (5-TAMRA), and a series of fluorescence quenching materials including gold nanoparticles (AuNPs), carbon nanotube (CNT), graphene oxide (GO), and thin-layer manganese dioxide (2D MnO_2_) were used for sensing.^20^ We envisioned that the presence of the quenching materials would lead to fluorescence quenching by generating supramolecular interactions with the fluorescent peptides, and the subsequent addition of viruses would lead to fluorescence recovery due to competitive binding between peptide ligands and RBDs.

The structure and purity of the fluorescently tagged peptides were confirmed by mass spectroscopy and high-performance liquid chromatography, respectively (see the Supporting Information). To analyze whether the conformation of the peptides is retained after truncation, circular dichroism (CD) was used (Figure S1). In their representative CD spectra, we observed two minima at 202 and 222 nm, characteristic of α-helices, for **QQ19**, **QI26**, and **LQ26**. In contrast, no typical signature of secondary structures was seen for **LL7** and **GI7**. This suggests that the relatively longer 19-mer truncated from the α_1_-helical sequence retained a helical structure. We also used AlphaFold2 to gain more structural information on the peptides.^21^ The results agree well with those obtained from CD that α-helical structures were putatively obtained for **QQ19**, **QI26**, and **LQ26**, and random coils obtained for **LL7** and **GI7** (Figure 2B).

### Construction and Characterization of The Peptide-Based Sensor Array

To construct the sensor array, we exploited a set of known quenching materials including AuNPs, CNTs, GO, and 2D MnO_2_. These low-dimensional materials have been well-established to quench the fluorescence of small-molecule dyes that are adsorbed to their surfaces.^22,23^ Indeed, mixing the fluorescent peptide probes with the materials led to concentration-dependent fluorescence quenching in phosphate buffered saline (PBS; 0.01 M, pH 7.4) (Figures S2–S5). The binding constants (*K*_a_) of different peptide probes with the materials were determined using the Benesi–Hildebrand eq (Table S1). To characterize the assembly between the peptides and materials, a series of techniques including high-resolution transmission electron microscopy (HR-TEM), zeta potential analysis, and Raman spectroscopy were used. Then, the peptide probe **TI26** with the strongest quenching effect was selected for characterization.

**Figure 3:**
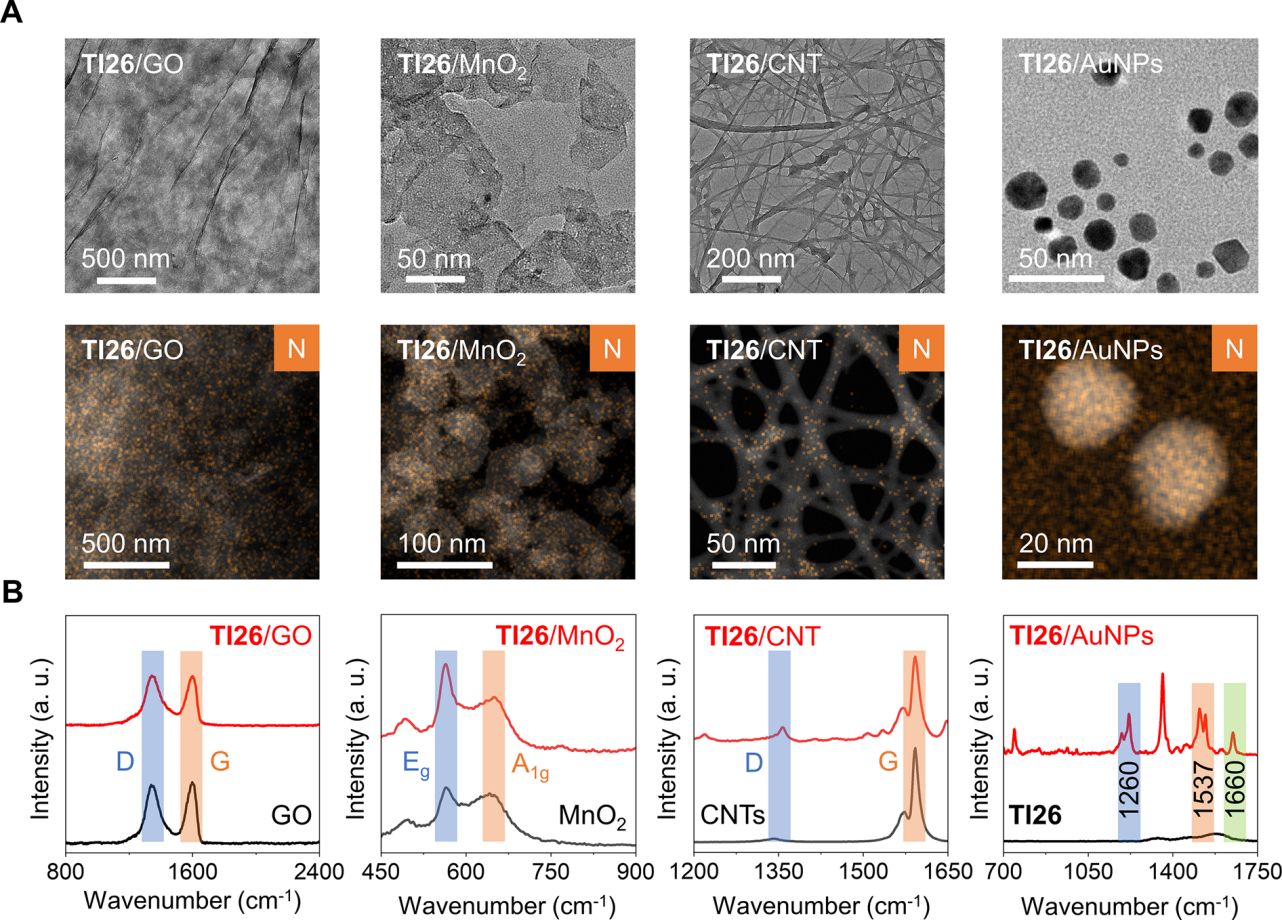
Characterization of peptide-material assembly. (A) HR-TEM and EDX-mapping images, and (B) Raman spectra of the ensembles formed between **TI26** and various quenching materials [**TI26**/GO (1 μM/7.5 μg mL^–1^), **TI26**/MnO_2_ (1 μM/6 μg mL^–1^), **TI26**/CNTs (1 μM/4 μg mL^–1^), **TI26**/AuNPs (1 μM/10 μg mL^–1^), GO (7.5 μg mL^–1^), MnO_2_ (6 μg mL^–1^), CNTs (1 μM/4 μg mL^–1^), AuNPs (1 μM/10 μg mL^–1^), and **TI26** (1 μM)].

In their representative HR-TEM images, we first observed morphologies typical for the low-dimensional materials used (Figure 3A and Figure S6). After assembly with **TI26**, dark-field TEM and the energy-dispersive X-ray spectroscopy (EDX) elemental mapping were used for the characterization (Figures S7–S10). We determined that the addition of the peptides did not cause the morphology of the materials to change. By mapping the nitrogen (N) element that does not exist in any of the quenching materials used, we observed an intense signal assigned to N for all **TI26**-material assemblies, suggesting the presence of peptide molecules on the surface of the materials (Figure 3A). The presence of **TI26** bearing the positively charged 5-TAMRA group also neutralized the negative zeta potentials of the low-dimensional materials (Figure S11).

Subsequently, Raman spectroscopy corroborated the binding between the peptide probes and materials (Figure 3B). For example, while minimal signals were detected for **TI26** alone, the presence of AuNPs significantly enhanced the Raman peak assigned to the C=O stretching (1660 cm^–1^), in-phase C–H/N–H stretching/bending (1537 cm^–1^), and out-of-phase C–H/N–H stretching/bending (1260 cm^–1^) of the peptide.^24^ For CNTs, a typical D band at ∼1350 cm^–1^ and a distinct G peak at 1590 cm^–1^ were observed with a D/G ratio of 0.04.^25^ After assembly with **TI26**, an increased D/G ratio of 0.19 was observed, suggesting a change in the defect level of CTNs upon peptide coating. From the Raman spectrum, GO exhibited characteristic D and G peaks at ∼1350 and ∼1585 cm^–1^, respectively, with a D/G ratio of 0.96. Assembly with the peptide probe increased the D/G ratio to 1.0, suggesting a defect change in the peptide-material assembly.^26^ Finally, wavenumbers found at 565 and 648 cm^–1^, characteristic of the E_g_ (Mn–O vibrations along the skeletal chain) and A_1g_ (symmetric stretching vibrations of Mn–O bonds), respectively, were observed for 2D MnO_2_ in its Raman spectrum. A corresponding E_g_/A_1g_ ratio of 1.11 was determined,^27^ and this value increased to 1.55 after complexation with **TI26**, suggesting the interruption of Mn–O bond vibrations.

### Use of The Fluorogenic Peptide Sensor Array for Classification of VOCs of SARS-Cov-2

With the sensor array constructed, we determined its capacity for SARS-Cov-2 classification. Due to safety concerns, we used pseudoviruses for the analyses. Based on the HIV-1 lentiviral packaging system, these pseudoviruses were prepared by cotransfecting cells with two plasmids—one expressing the SARS-CoV-2 S-protein and the other encoding the HIV-1 packaging protein and signaling with the envelope gene removed. With this protocol, peudoviruses bearing the S-protein from a wild-type (**WT**) strain isolated in Wuhan (GISAID EPI_ISL_402125) and five WHO-announced VOCs including B.1.1.7 (**Alpha**), B.1.351 (**Beta**), P.1 (**Gamma**), B.1.617.2 (**Delta**), and BA.2 (**Omicron**) were constructed and used for the classification; the VOCs are hereinafter referred to as their Greek alphabetic terms for clarity. A number of other viral species including a SARS-CoV pseudovirus, influenza A viruses (H1N1, H3N2, H7N9, H5N1 and H10N8), chimpanzee adenovirus serotype 68 (AdC68), human adenovirus serotype 7 (Ad7), a middle east respiratory syndrome (MERS) pseudovirus, a Marburg virus (MARV) pseudovirus, an Ebola virus (EBOV) pseudovirus, vesicular stomatitis virus (VSV), and rabies challenge virus standard 11 (CVS-11) were used as negative controls.

**Figure 4:**
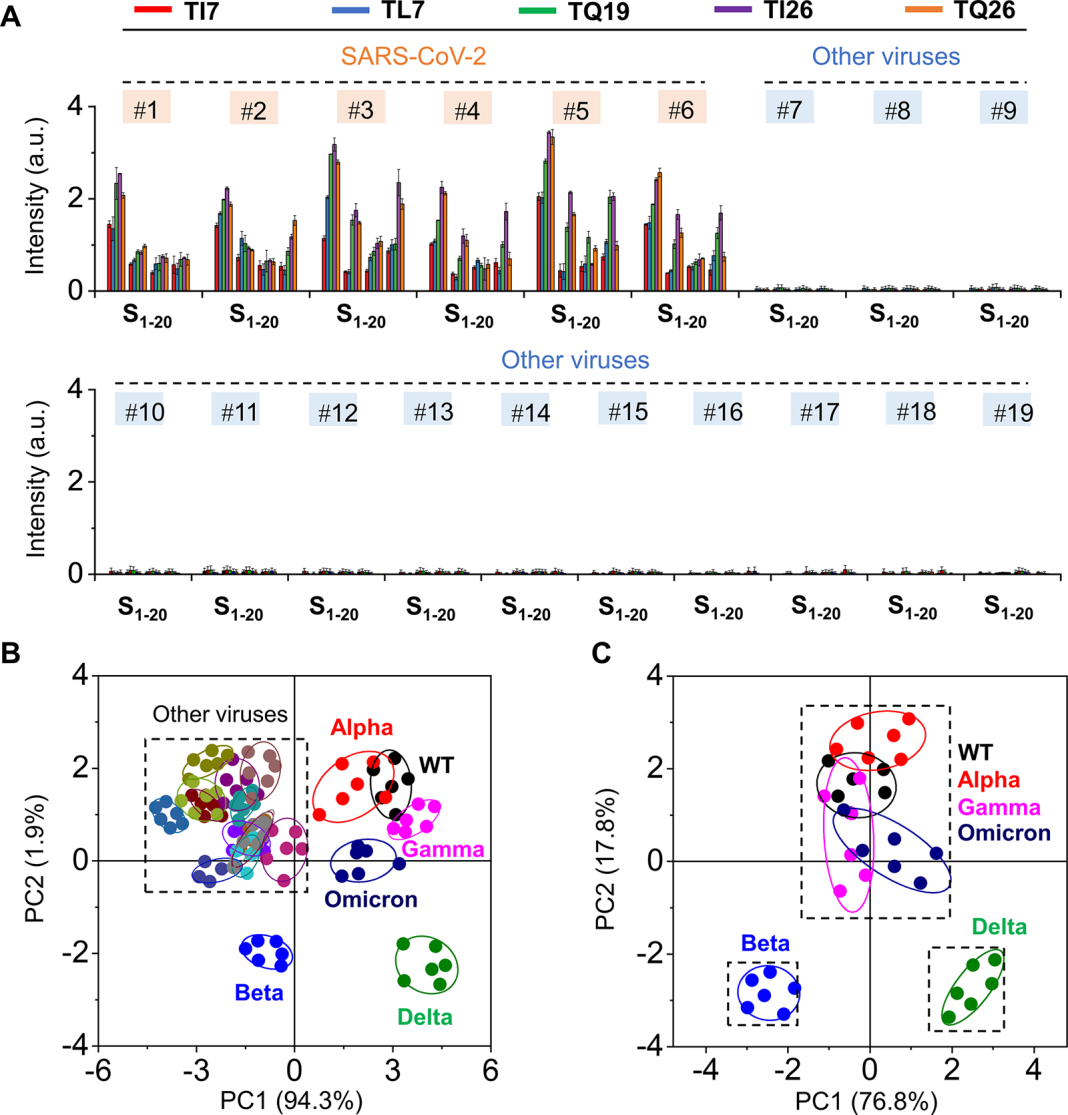
Use of the fluorogenic peptide sensor array for virus classification. (A) Fluorescence intensity changes of **S**_**1**_–**S**_**20**_ seen in the presence of SARS-CoV-2 and other viruses. (B) PCA of the fluorescence changes of the **S**_**1**_–**S**_**20**_ sensor array (See Table S2 for naming rules) to differentiate SARS-CoV-2 from other viruses. (C) PCA of the fluorescence changes of the **S**_**1**_–**S**_**20**_ sensor array to classify VOCs of SARS-CoV-2. The viruses used are #1: **WT**, #2: **Alpha**, #3: **Beta**, #4: **Gamma**, #5: **Delta**, #6: **Omicron**, #7: SARS-CoV, #8: H1N1, #9: H3N2, #10: H7N9, #11: H5N1, #12: H10N8, #13: MERS, #14: MARV, #15: AdC68, #16: Ad7, #17: EBOV, #18: VSV, and #19: CVC-11. All measurements were done on a M5 microplate reader in PBS (0.01 M, pH 7.4); λ_em_ = 560 nm, λ_ex_ = 520 nm.

The peptide-material ensembles were orthogonally incubated with the viruses detailed above in a 384-well plate, and then, the fluorescence signals (λ_560_ upon excitation at 520 nm) generated were recorded by a fluorescence microplate reader (Molecular Device M5, USA) in a high-throughput manner. The fluorescence signals were further processed by PCA to create a “fingerprint” corresponding to each viral analyte.^28^ We found that the presence of SARS-CoV-2 pseudoviruses elicited a much stronger fluorescence recovery of the quenched peptide probes than other control viruses (Figure 4A). This implies selective binding of the hACE2-derived peptides with RBDs on the surface of the viruses. Through PCA plotting, we determined that the fingerprints of SARS-CoV-2 pseudoviruses and those of other viruses were situated to the right and left halves, respectively, with an exception for **Beta**, which is solely plotted to the bottom-left quadrant (Figure 4B). This preliminarily suggests the effectiveness of the sensor array in distinguishing SARS-CoV-2 from other viruses including SARS-CoV, another β-coronavirus that shares a 76% identity in S-protein sequence with SARS-CoV-2, which also interacts with hACE2 to enter cells.^29^

An analysis in more detail for the SARS-CoV-2 pseudoviral strains indicated a good separation of the fingerprints of **Beta** and **Delta** variants from those of **WT**, **Alpha**, **Gamma**, and **Omicron** (Figure 4C). We then sought to rationalize this differentiation by referring to the genetic information on each SARS-CoV-2 strain. The phylogenetic tree of SARS-CoV-2 genome sequences from **WT** to **Omicron** indicated that the VOCs could be divided into three branches, in which one branch includes **Alpha**, **Gamma**, and **Omicron** with closer evolutionary relationships, and the other two branches include **Beta** and **Delta**, respectively (Figure S13).^30^ This implies that the classification result obtained from our sensor array roughly matches the phylogenetic analysis of the VOCs. More interestingly, a recent study also indicated a stronger infectivity of **Beta** and **Delta** than those of **Alpha**, **Gamma**, and **Omicron**.^31^ These results prompted us to further scrutinize peptide-RBD binding at the molecular level.

**Figure 5:**
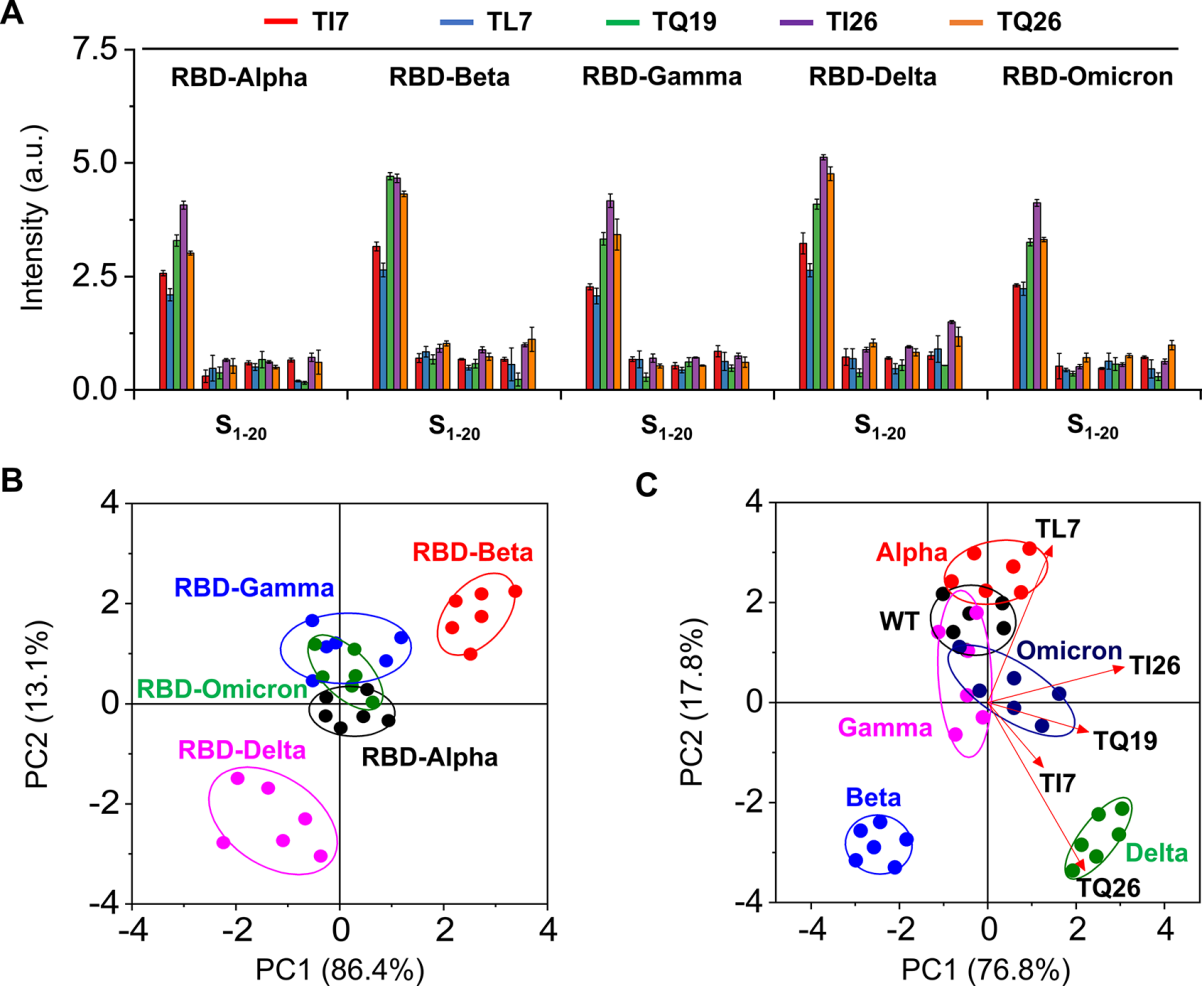
Use of the fluorogenic peptide sensor array for RBDs. (A) Fluorescence intensity changes of **S**_**1**_–**S**_**20**_ seen in the presence of VOC RBDs. (B) PCA of the fluorescence changes of the **S**_**1**_–**S**_**20**_ sensor array for RBDs from the VOCs. (C) PCA and loading plots of the fluorescence changes of the **S**_**1**_–**S**_**20**_ sensor array for VOCs. All measurements were done on a M5 microplate reader in PBS (0.01 M, pH 7.4); λ_em_ = 560 nm, λ_ex_ = 520 nm.

We expressed the RBDs from the S-proteins of the five VOCs according to their established sequences and used our sensor array for classification. We observed that the presence of **Beta** or **Delta** elicited a stronger fluorescence recovery of the quenched peptide probes than other VOCs (Figure 5A and Figure S14). Shown in Figure 5B is the classification result, which agrees well with those obtained for the VOC pseudoviruses (Figure 4B). This suggests that the peptide-RBD binding predominantly determines the array outcome. We also used fluorescence spectroscopy to evaluate the binding. The peptide-GO complexes exhibiting the strongest fluorescence responses in the microplate-reader assay were employed. We found that the presence of the RBDs caused the fluorescence of the complexes to recover in a concentration-dependent manner (Figure S15). The detection limits of the systems for the RBDs were then determined from the fluorescence data (Figure S16), indicating that the sensitivity of the peptide probes for the RBDs is in an order of **TI26** > **TQ26** > **TQ19** > **TL7** > **TI7**. This agrees with the loading plots produced for Figure 5C that the contribution of peptide probes for VOC classification is in the same order of **TI26** > **TQ26** > **TQ19** > **TL7** > **TI7**.

To validate the PCA results, we also used other analytical techniques including linear discriminant analysis (LDA) and hierarchical clustering analysis (HCA) to process the data from the high-throughput array. The results, shown in Figures S17–S19, indicated that the outcomes obtained using LDA and HCA are consistent with those obtained using PCA, supporting the reliability of our system.

We also carried out a fluorescence titration using **TI26**/MnO_2_ with different VOCs (Figure S20) to determine the binding constant between the peptide and viruses (*K*_a_) through the Benesi–Hildebrand eq (Figure S21). The results indicated that the *K*_a_ of **TI26**/MnO_2_ for different VOCs is in an order of **Beta** > **Delta** > **Alpha** > **Gamma** > **Omicron** > **WT**. Among them, the *K*_a_ of the peptide ligand for **Alpha**, **Gamma**, and **Omicron** was closer to that for **WT**. This corroborated that the binding affinity between the peptide ligands and RBDs determines the fluorescence recovery rate, which in turn affects the classification results of the PCA.

Finally, we optimized the sensor array by determining the minimum number of hosts (peptide-material ensembles) required to effectively differentiate the VOCs. The loading plots produced (Figure 4C) provided information on which peptide probe (Figure 5C) and material (Figure S22) contributed the most to the classification. An analysis by reducing the number of materials used in the sensor array indicated that a combination of 2D MnO_2_, AuNPs, and GO differentiated the viral strains, but further elimination of GO significantly compromised the analytical result (Figure S23). Subsequently, we determined that a combination of just **TI26**, **TQ26**, and **TQ19** led to **Beta** and **Delta** being well-differentiated from other variants; however, the fingerprints for **WT**, **Alpha**, **Gamma**, and **Omicron** became more convergent (Figure S24). Again, the further elimination of **TQ19** compromised the classification, suggesting that the sensor array required a combination of at least three peptide probes to achieve sufficient differentiation of VOCs.

## Conclusions

In this study, we have developed a rationally designed fluorogenic peptide array for the classification of VOCs of SARS-CoV-2. The rationale by which the array was constructed relies on the truncation of a series of key binding peptides from the human receptor of the viral RBD to allow the generation of moderate host–guest interactions. Upon mutation, the binding behavior of the peptides for the mutated variants varies, which in turn produces fingerprints characteristic of each viral strain through PCA. We confirmed that the sensor array accurately differentiates WHO-announced VOCs of SARS-CoV-2 pseudoviruses as well as recombinant RBDs, and the results agree well with those obtained from analyses of the evolutionary relationship of the viruses.

This research may help guide the development of effective sensing tools for the timely and accurate classification of VOCs arising from acutely contagious viruses using simple high-throughput assays and employing readily available instrumentation (e.g., a microplate reader). We anticipate that the concept of using truncated peptides from the binding region of a viral protein and human receptor will facilitate research into such host–guest interactions in the future.^32−35^ Using our approach, it is possible to easily construct a sensor array system for any target virus, by simply introducing a fluorescent tag to the truncated peptides, which can then be used as part of an array in combination with quenching materials. Subsequent data processing using dimensionality reduction techniques such as PCA then facilitates the rapid classification of the target virus in the presence of mutants.

## Supporting Information Available

Additional experimental details, materials and methods, Figures S1–S24, original MS and HPLC of peptides (Figures S25–S44), and Tables S1 and S2 (PDF)
