# Supplementary material for: Fluorogenic Peptide Sensor Array Derived from Angiotensin-Converting Enzyme 2 Classifies Severe Acute Respiratory Syndrome Coronavirus 2 Variants of Concern
Source: J Am Chem Soc. 2024 Jul 19;146(30):21017–24. doi: 10.1021/jacs.4c06172 (PMC11295173; doi:10.1021/jacs.4c06172)
Supplement: Supplementary file 1 — ja4c06172_si_001.pdf [file ja4c06172_si_001.pdf]

Supporting Information

Fluorogenic Peptide Sensor Array Derived from Angiotensin-Converting Enzyme 2 Classifies Severe Acute Respiratory Syndrome Coronavirus 2 Variants of Concern

Wei-Tao Dou[a#], Pei-Hong Tong[a#], Man Xing[d#], Jiao-Jiao Liu[d], Xi-Le Hu[a], Tony D James[e,f]*, Dong-Ming Zhou[c,d]*, & Xiao-Peng He[a,b]*

[a] *Key Laboratory for Advanced Materials and Joint International Research Laboratory of Precision Chemistry and Molecular Engineering, Feringa Nobel Prize Scientist Joint Research Center, Frontiers Center for Materiobiology and Dynamic Chemistry, School of Chemistry and Molecular Engineering, East China University of Science and Technology, 130 Meilong Rd., Shanghai 200237, China.*

[b] *The International Cooperation Laboratory on Signal Transduction, National Center for Liver Cancer, Eastern Hepatobiliary Surgery Hospital, Shanghai 200438, China.*

[c] *Vaccine and Immunity Research Center, Shanghai Public Health Clinical Center, Fudan University, Shanghai, China.*

[d] *Department of Pathogen Biology, School of Basic Medical Sciences, Tianjin Medical University, Tianjin, China.*

[e] *Department of Chemistry, University of Bath, Bath, BA2 7AY, UK.*

[f] *School of Chemistry and Chemical Engineering, Henan Normal University, Xinxiang 453007, China.*

[#]These authors contributed equally.

***Corresponding authors.** xphe@ecust.edu.cn; zhoudongming@tmu.edu.cn; t.d.james@bath.ac.uk

Contents list

S1. Experimental procedures
S2. Additional figures
S3. Table
S4. Additional references

S1. Experimental procedures

General. All purchased chemicals and reagents were of analytical grade. Receptor-binding domain (RBD) of the S proteins **(RBD-Alpha**, **RBD-Beta**, **RBD-Delta**, **RBD-Gamma** and **RBD-Omicron)** were customized from Beijing BioNC Biotech Co., Ltd. High-resolution transmission electron microscope (HRTEM) images were obtained with a Talos F200X instrument equipped with a Gatan Orius charged-coupled device camera and a Tridiem energy filter operating at 200 kV. Zeta potential was carried out on a Horiba LB-550 Dynamic Light Scattering Nano-Analyzer. Raman spectra were obtained using a Renishaw InVia Reflex Raman system (Renishaw plc, Wotton-under-Edge, UK) employing a grating spectrometer with a Peltier-cooled charge-coupled device (CCD) detector coupled to a confocal microscope, which were then processed with Renishaw WiRE 3.2 software. The Raman scattering was excited by an argon ion laser (I = 514.5 nm). Fluorescence spectra were recorded on a Varian Cary Eclipse fluorescence spectrophotometer. The sensor array experiments were carried out using an M5 microplate reader (Molecular Device, USA) or DR-3000 microplate reader (Diatek, China) with an excitation wavelength of 520 nm.

Synthesis of peptide ligands LL-7, GI-7, LQ-26, QI-26 and QQ-19, TL-7, TI-7, TQ-26, TI-26 and TQ-19. Peptides were synthesized by the solid-phase method using reagent systems and methodologies of standard Fmoc-chemistry.[1]

GI-7: Calculated mass for $C_{35}H_{58}N_{11}O_{10}^{+}$ $[M + H]^{+}$: 792.43, found (MALDI-TOF): $[M + H]^{+}$: 792.55. HPLC: t_R= 9.957 min over 30 min of 1 mL min^{-1} mobile phase (17% solvent A (0.1% trifluoroacetic in 100% acetonitrile) and 83% solvent B (0.1% trifluoroacetic in 100% Water)), purity 96.03% (Figures S25 and S26).

LL-7: Calculated mass for $C_{39}H_{63}N_{8}O_{10}S^{+}$ $[M + H]^{+}$: 835.44, found (MALDI-TOF): $[M + H]^{+}$: 835.50. HPLC: t_R= 9.980 min over 30 min of 1 mL min^{-1} mobile phase (17% solvent A (0.1% trifluoroacetic in 100% acetonitrile) and 83% solvent B (0.1% trifluoroacetic in 100% Water)), purity 96.73% (Figures S27 and S28).

LQ-26: Calculated mass for $C_{147}H_{217}N_{34}O_{42}S^{+}$ $[M + 3H]^{3+}$: 1054.85, found (MALDI-TOF): $[M + 3H]^{3+}$: 1054.80 HPLC: t_R= 11.205 min over 30 min of 1 mL min^{-1} mobile phase (27% solvent A (0.1% trifluoroacetic in 100% acetonitrile) and 73% solvent B (0.1% trifluoroacetic in 100% Water)), purity 96.92% (Figures S29 and S30).

QI-26: Calculated mass for $C_{143}H_{214}N_{37}O_{42}^{+}$ $[M + 5H]^{5+}$: 624.69, found (MALDI-TOF): $[M + 5H]^{5+}$: 624.65 HPLC: t_R= 9.283 min over 30 min of 1 mL min^{-1} mobile phase (25% solvent A (0.1% trifluoroacetic in 100% acetonitrile) and 75% solvent B (0.1% trifluoroacetic in 100% Water)), purity 96.14% (Figures S31 and S32).

QQ-19: Calculated mass for $C_{108}H_{156}N_{26}O_{33}^+$ $[M + 2H]^{2+}$: 1173.27, found (MALDI-TOF): $[M + 2H]^{2+}$: 1173.20 HPLC: t_R= 11.283 min over 30 min of 1 mL min^{-1} mobile phase (23% solvent A (0.1% trifluoroacetic in 100% acetonitrile) and 77% solvent B (0.1% trifluoroacetic in 100% Water)), purity 96.45% (Figures S33 and S34).

TL-7: Calculated mass for $C_{64}H_{83}N_{10}O_{14}S^+$ $[M + 2H]^{2+}$: 624.75, found (MALDI-TOF): $[M + 2H]^{2+}$: 624.5. HPLC: t_R= 11.851 min over 30 min of 1 mL min^{-1} mobile phase (65% solvent A (0.1% trifluoroacetic in 100% acetonitrile) and 35% solvent B (0.1% trifluoroacetic in 100% Water)), purity 96.86% (Figures S35 and S36).

TI-7: Calculated mass for $C_{60}H_{78}N_{13}O_{14}^+$ $[M + 3H]^{3+}$: 402.46, found (MALDI-TOF): $[M + 3H]^{3+}$: 402.5. HPLC: t_R= 12.059 min over 30 min of 1 mL min^{-1} mobile phase (50% solvent A (0.1% trifluoroacetic in 100% acetonitrile) and 50% solvent B (0.1% trifluoroacetic in 100% Water)), purity 95.48% (Figures S37 and S38).

TQ-26: Calculated mass for $C_{172}H_{235}N_{36}O_{46}S^+$ $[M + 3H]^{3+}$: 1192.36, found (MALDI-TOF): $[M + 3H]^{3+}$: 1192.5. HPLC: t_R= 12.742 min over 30 min of 1 mL min^{-1} mobile phase (62% solvent A (0.1% trifluoroacetic in 100% acetonitrile) and 38% solvent B (0.1% trifluoroacetic in 100% Water)), purity 95.24% (Figures S39 and S40).

TI-26: Calculated mass for $C_{168}H_{230}N_{39}O_{46}^+$ $[M + 5H]^{5+}$: 707.19, found (MALDI-TOF): $[M + 5H]^{5+}$: 707.3. HPLC: t_R= 11.715 min over 30 min of 1 mL min^{-1} mobile phase (55% solvent A (0.1% trifluoroacetic in 100% acetonitrile) and 45% solvent B (0.1% trifluoroacetic in 100% Water)), purity 95.29% (Figures S41 and S42).

TQ-19: Calculated mass for $C_{133}H_{175}N_{28}O_{37}^+$ $[M + 4H]^{4+}$: 690.27, found (MALDI-TOF): $[M + 4H]^{4+}$: 690.3. HPLC: t_R= 10.635 min over 30 min of 1 mL min^{-1} mobile phase (57% solvent A (0.1% trifluoroacetic in 100% acetonitrile) and 43% solvent B (0.1% trifluoroacetic in 100% Water)), purity 95.36% (Figures S43 and S44).

Structure prediction. AlphaFold structure predictions were performed using a locally installed copy of the ColabFold-batch 1.5. program, giving as input only the amino acid sequence of peptides. Predictions were run for peptide structures of **LL-7**, **GI-7**, **QQ-19**, **LQ-26**, and of **QI-26**. For each entry, five structures were generated with AlphaFold 2 and the best-ranking structures according to the LDDT scores were selected as the representative pose for the entry.[2,3] Images of proteins are visualized using PyMol.

Circular dichroism spectra. The concentration of the peptides tested was 0.2 mg mL^{-1}. Using a 1 mm quartz cuvette, circular dichroism spectra of the five peptides were recorded from a spectral range of 190 to 240 nm on a Jasco J-815 spectrophotometer at 25 °C. The scanning speed was 50

nm min^{-1} with a bandwidth of 1 nm. The spectrum of the sample buffer was subtracted as blank control.

Preparation of SARS-CoV-2 pseudoviruses. HEK 293T cells were co-transfected with HIV-1 backbone plasmid pNL4-3.Luc.R-E and pCAGGS-S-CΔ19 expressing SARS-CoV-2 S-protein with a deletion of 19-amino acids from the C-terminus. The S-protein amino acid sequences of the **WT** strain and its variants were based on GISAID EPI_ISL_402125 (**WT** strain), EPI_ISL_810967 (B.1.1.7 variant), EPI_ISL_18336869 (B.1.351 variant), EPI_ISL_2443693 (P.1 variant), EPI_ISL_2788592 (B.1.617.2 variant), or EPI_ISL_19031860 (BA.2 variant). The supernatant was harvested 48 hours after transfection and filtered through 0.45 μm filters. After overnight incubation with PEG8000 at 4 °C and centrifugation at 3000 × g for 30 min, the pseudovirus particles were concentrated and purified. Aliquoted pseudovirus particles were stored at -80 °C until further use. For pseudovirus titration, lentivirus quantitation kit (Beijing Biodragon Immunotechnologies Co., Ltd, Beijing, China) was used following the manufacturer's instructions.

Annotation: The SARS-CoV-2 pseudovirus is a recombinant virus that has lentivirus-derived backbone proteins along with surface proteins obtained from SARS-CoV-2. The conformational structures of pseudoviral surface S proteins have high similarity to that of the native viral proteins, but such pseudoviruses do not contain the SARS-CoV-2 genome as well as being replication-deficient, which offers a safer alternative for studying SARS-CoV-2. Therefore, pseudovirus is widely employed in research on functions and processes related to S protein, such as cellular tropism[4], receptor recognition[5], drug screening[6] and antibody evaluation[7].

>*Original strain*
MFVFLVLLPLVSSQCVNLTTRTQLPPAYTNSFTRGVYYPDKVFRSSVLHSTQDLFLPFFS
NVTWFHAIHVSGTNGTKRFDNPVLPFNDGVYFASTEKSNIIRGWIFGTTLDSKTQSLLIV
NNATNVVIKVCEFQFCNDPFLGVYYHKNNKSWMESEFRVYSSANNCTFEYVSQPFLMD
LEGKQGNFKNLREFVFKNIDGYFKIYSKHTPINLVRDLPQGFSALEPLVDLPIGINITRFQT
LLALHRSYLTPGDSSSGWTAGAAAYYVGYLQPRTFLLKYNENGTITDAVDCALDPLSET
KCTLKSFTVEKGIYQTSNFRVQPTESIVRFPNITNLCPFGEVFNATRFASVYAWNRKRISN
CVADYSVLYNSASFSTFKCYGVSPTKLNDLCFTNVYADSFVIRGDEVRQIAPGQTGKIA
DYNYKLPDDFTGCVIAWNSNNLDSKVGGNYNYLYRLFRKSNLKPFERDISTEIYQAGST
PCNGVEGFNCYFPLQSYGFQPTNGVGYQPYRVVVLSFELLHAPATVCGPKKSTNLVKN
KCVNFNFNGLTGTGVLTESNKKFLPFQQFGRDIADTTDAVRDPQTLEILDITPCSFGGVS
VITPGTNTSNQVAVLYQDVNCTEVPVAIHADQLTPTWRVYSTGSNVFQTRAGCLIGAEH
VNNSYECDIPIGAGICASYQTQTNSPRRARSVASQSIIAYTMSLGAENSVAYSNNSIAIPT
NFTISVTTEILPVSMTKTSVDCTMYICGDSTECSNLLLQYGSFCTQLNRALTGIAVEQDK
NTQEVFAQVKQIYKTPPIKDFGGFNFSQILPDPSKPSKRSFIEDLLFNKVTLADAGFIKQY

GDCLGDIAARDLICAQKFNGLTVLPPLLTDEMIAQYTSALLAGTITSGWTFGAGAALQIP
FAMQMAYRFNGIGVTQNVLYENQKLIANQFNSAIGKIQDSLSSTASALGKLQDVVNQN
AQALNTLVKQLSSNFGAISSVLNDILSRLDKVEAEVQIDRLITGRLQSLQTYVTQQLIRAA
EIRASANLAATKMSECVLGQSKRVDFCGKGYHLMSFPQSAPHGVVFLHVTYVPAQEKN
FTTAPAICHDGKAHFPREGVFVSNGTHWFVTQRNFYEPQIITTDNTFVSGNCDVVIGIVN
NTVYDPLQPELDSFKEELDKYFKNHTSPDVDLGDISGINASVVNIQKEIDRLNEVAKNLN
ESLIDLQELGKYEQYIKWPWYIWLGFIAGLIAIVMVTIMLCCMTSCCSCLKGCCSCGSCC
KFDEDDSEPVLKGVKLHYT*

>B.1.1.7 variant
MFVFLVLLPLVSSQCVNLTTRTQLPPAYTNSFTRGVYYPDKVFRSSVLHSTQDLFLPFFS
NVTWFHAISGTNGTKRFDNPVLPFNDGVYFASTEKSNIIRGWIFGTTLDSKTQSLLIVNN
ATNVVIKVCEFQFCNDPFLGVYHKNNKSWMESEFRVYSSANNCTFEYVSQPFLMDLEG
KQGNFKNLREFVFKNIDGYFKIYSKHTPINLVRDLPQGFSALEPLVDLPIGINITRFQTLLA
LHRSYLTPGDSSSGWTAGAAAYYVGYLQPRTFLLKYNENGTITDAVDCALDPLSETKCT
LKSFTVEKGIYQTSNFRVQPTESIVRFPNITNLCPFGEVFNATRFASVYAWNRKRISNCVA
DYSVLYNSASFSTFKCYGVSPTKLNDLCFTNVYADSFVIRGDEVRQIAPGQTGKIADYN
YKLPDDFTGCVIAWNSNNLDSKVGGNYNYLYRLFRKSNLKPFERDISTEIYQAGSTPCN
GVEGFNCYFPLQSYGFQPTYGVGYQPYRVVVLSFELLHAPATVCGPKKSTNLVKNKCV
NFNFNGLTGTGVLTESNKKFLPFQQFGRDIDDTTDAVRDPQTLEILDITPCSFGGVSVITP
GTNTSNQVAVLYQGVNCTEVPVAIHADQLTPTWRVYSTGSNVFQTRAGCLIGAEHVNN
SYECDIPIGAGICASYQTQTNSHRRARSVASQSIIAYTMSLGAENSVAYSNNSIAIPINFTIS
VTTEILPVSMTKTSVDCTMYICGDSTECSNLLLQYGSFCTQLNRALTGIAVEQDKNTQEV
FAQVKQIYKTPPIKDFGGFNFSQILPDPSKPSKRSFIEDLLFNKVTLADAGFIKQYGDCLG
DIAARDLICAQKFNGLTVLPPLLTDEMIAQYTSALLAGTITSGWTFGAGAALQIPFAMQM
AYRFNGIGVTQNVLYENQKLIANQFNSAIGKIQDSLSSTASALGKLQDVVNQNAQALNT
LVKQLSSNFGAISSVLNDILARLDKVEAEVQIDRLITGRLQSLQTYVTQQLIRAAEIRASA
NLAATKMSECVLGQSKRVDFCGKGYHLMSFPQSAPHGVVFLHVTYVPAQEKNFTTAPA
ICHDGKAHFPREGVFVSNGTHWFVTQRNFYEPQIITTHNTFVSGNCDVVIGIVNNTVYDP
LQPELDSFKEELDKYFKNHTSPDVDLGDISGINASVVNIQKEIDRLNEVAKNLNESLIDLQ
ELGKYEQYIKWPWYIWLGFIAGLIAIVMVTIMLCCMTSCCSCLKGCCSCGSCCKFDEDD
SEPVLKGVKLHYT*

>B.1.351 variant
MFVFLVLLPLVSSQCVNFTTRTQLPPAYTNSFTRGVYYPDKVFRSSVLHSTQDLFLPFFS
NVTWFHAIHVSGTNGTKRFANPVLPFNDGVYFASTEKSNIIRGWIFGTTLDSKTQSLLIV
NNATNVVIKVCEFQFCNDPFLGVYYHKNNKSWMESEFRVYSSANNCTFEYVSQPFLMD
LEGKQGNFKNLREFVFKNIDGYFKIYSKHTPINLVRGLPQGFSALEPLVDLPIGINITRFQT
LHRSYLTPGDSSSGWTAGAAAYYVGYLQPRTFLLKYNENGTITDAVDCALDPLSETKCT

LKTFTVEKGIYQTSNFRVQPTESIVRFPNITNLCPFGEVFNATRFASVYAWNRKRISNCVA
DYSVLYNSASFSTFKCYGVSPTKLNDLCFTNVYADSFVIRGDEVRQIAPGQTGNIADYN
YKLPDDFTGCVIAWNSNNLDSKVGGNYNYLYRLFRKSNLKPFERDISTEIYQAGSTPCN
GVKGFNCYFPLQSYGFQPTYGVGYQPYRVVVLSFELLHAPATVCGPKKSTNLVKNKCV
NFNFNGLTGTGVLTESNKKFLPFQQFGRDIADTTDAVRDPQTLEILDITPCSFGGVSVITP
GTNTSNQVAVLYQGVNCTEVPVAIHADQLTPTWRVYSTGSNVFQTRAGCLIGAEHVNN
SYECDIPIGAGICASYQTQTNSPRRARSVASQSIIAYTMSLGVENSVAYSNNSIAIPTNFTIS
VTTEILPVSMTKTSVDCTMYICGDSTECSNLLLQYGSFCTQLNRALTGIAVEQDKNTQEV
FAQVKQIYKTPPIKDFGGFNFSQILPDPSKPSKRSFIEDLLFNKVTLADAGFIKQYGDCLG
DIAARDLICAQKFNGLTVLPPLLTDEMIAQYTSALLAGTITSGWTFGAGAALQIPFAMQM
AYRFNGIGVTQNVLYENQKLIANQFNSAIGKIQDSLSSTASALGKLQDVVNQNAQALNT
LVKQLSSNFGAISSVLNDILSRLDKVEAEVQIDRLITGRLQSLQTYVTQQLIRAAEIRASA
NLAATKMSECVLGQSKRVDFCGKGYHLMSFPQSAPHGVVFLHVTYVPAQEKNFTTAPA
ICHDGKAHFPREGVFVSNGTHWFVTQRNFYEPQIITTDNTFVSGNCDVVIGIVNNTVYDP
LQPELDSFKEELDKYFKNHTSPDVDLGDISGINASVVNIQKEIDRLNEVAKNLNESLIDLQ
ELGKYEQYIKWPWYIWLGFIAGLIAIVMVTIMLCCMTSCCSCLKGCCSCGSCCKFDEDD
SEPVLKGVKLHYT*

>P.1 variant

MFVFLVLLPLVSSQCVNFTNRTQLPSAYTNSFTRGVYYPDKVFRSSVLHSTQDLFLPFFS
NVTWFHAIHVSGTNGTKRFDNPVLPFNDGVYFASTEKSNIIRGWIFGTTLDSKTQSLLIV
NNATNVVIKVCEFQFCNYPFLGVYYHKNNKSWMESEFRVYSSANNCTFEYVSQPFLMD
LEGKQGNFKNLSEFVFKNIDGYFKIYSKHTPINLVRDLPQGFSALEPLVDLPIGINITRFQT
LLALHRSYLTPGDSSSGWTAGAAAYYVGYLQPRTFLLKYNENGTITDAVDCALDPLSET
KCTLKSFTVEKGIYQTSNFRVQPTESIVRFPNITNLCPFGEVFNATRFASVYAWNRKRISN
CVADYSVLYNSASFSTFKCYGVSPTKLNDLCFTNVYADSFVIRGDEVRQIAPGQTGTIAD
YNYKLPDDFTGCVIAWNSNNLDSKVGGNYNYLYRLFRKSNLKPFERDISTEIYQAGSTP
CNGVKGFNCYFPLQSYGFQPTYGVGYQPYRVVVLSFELLHAPATVCGPKKSTNLVKNK
CVNFNFNGLTGTGVLTESNKKFLPFQQFGRDIADTTDAVRDPQTLEILDITPCSFGGVSVI
TPGTNTSNQVAVLYQGVNCTEVPVAIHADQLTPTWRVYSTGSNVFQTRAGCLIGAEYV
NNSYECDIPIGAGICASYQTQTNSPRRARSVASQSIIAYTMSLGAENSVAYSNNSIAIPTNF
TISVTTEILPVSMTKTSVDCTMYICGDSTECSNLLLQYGSFCTQLNRALTGIAVEQDKNT
QEVFAQVKQIYKTPPIKDFGGFNFSQILPDPSKPSKRSFIEDLLFNKVTLADAGFIKQYGD
CLGDIAARDLICAQKFNGLTVLPPLLTDEMIAQYTSALLAGTITSGWTFGAGAALQIPFA
MQMAYRFNGIGVTQNVLYENQKLIANQFNSAIGKIQDSLSSTASALGKLQDVVNQNAQ
ALNTLVKQLSSNFGAISSVLNDILSRLDKVEAEVQIDRLITGRLQSLQTYVTQQLIRAAEI
RASANLAAIKMSECVLGQSKRVDFCGKGYHLMSFPQSAPHGVVFLHVTYVPAQEKNFT
TAPAICHDGKAHFPREGVFVSNGTHWFVTQRNFYEPQIITTDNTFVSGNCDVVIGIVNNT

VYDPLQPELDSFKEELDKYFKNHTSPDVDLGDISGINASSVNIQKEIDRLNEVAKNLNES
LIDLQELGKYEQYIKWPWYIWLGFIAGLIAIVMVTIMLCCMTSCCSCLKGCCSCGSCCKF
DEDDSEPVLKGVKLHYT*

>B.1.617.2 variant
MFVFLVLLPLVSSQCVNLRTRTQLPPAYTNSFTRGVYYPDKVFRSSVLHSTQDLFLPFFS
NVTWFHAIHVSGTNGTKRFDNPVLPFNDGVYFASTEKSNIIRGWIFGTTLDSKTQSLLIV
NNATNVVIKVCEFQFCNDPFLDVYYHKNNKSWMESGVYSSANNCTFEYVSQPFLMDLE
GKQGNFKNLREFVFKNIDGYFKIYSKHTPINLVRDLPQGFSALEPLVDLPIGINITRFQTLL
ALHRSYLTPGDSSSGWTAGAAAYYVGYLQPRTFLLKYNENGTITDAVDCALDPLSETK
CTLKSFTVEKGIYQTSNFRVQPTESIVRFPNITNLCPFGEVFNATRFASVYAWNRKRISNC
VADYSVLYNSASFSTFKCYGVSPTKLNDLCFTNVYADSFVIRGDEVRQIAPGQTGKIAD
YNYKLPDDFTGCVIAWNSNNLDSKVGGNYNYRYRLFRKSNLKPFERDISTEIYQAGSKP
CNGVEGFNCYFPLQSYGFQPTNGVGYQPYRVVVLSFELLHAPATVCGPKKSTNLVKNK
CVNFNFNGLTGTGVLTESNKKFLPFQQFGRDIADTTDAVRDPQTLEILDITPCSFGGVSVI
TPGTNTSNQVAVLYQGVNCTEVPVAIHADQLTPTWRVYSTGSNVFQTRAGCLIGAEHV
NNSYECDIPIGAGICASYQTQTNSRRRARSVASQSIIAYTMSLGAENSVAYSNNSIAIPTNF
TISVTTEILPVSMTKTSVDCTMYICGDSTECSNLLLQYGSFCTQLNRALTGIAVEQDKNT
QEVFAQVKQIYKTPPIKDFGGFNFSQILPDPSKPSKRSFIEDLLFNKVTLADAGFIKQYGD
CLGDIAARDLICAQKFNGLTVLPPLLTDEMIAQYTSALLAGTITSGWTFGAGAALQIPFA
MQMAYRFNGIGVTQNVLYENQKLIANQFNSAIGKIQDSLSSTASALGKLQNVVNQNAQ
ALNTLVKQLSSNFGAISSVLNDILSRLDKVEAEVQIDRLITGRLQSLQTYVTQQLIRAAEI
RASANLAATKMSECVLGQSKRVDFCGKGYHLMSFPQSAPHGVVFLHVTYVPAQEKNF
TTAPAICHDGKAHFPREGVFVSNGTHWFVTQRNFYEPQIITTDNTFVSGNCDVVIGIVNN
TVYDPLQPELDSFKEELDKYFKNHTSPDVDLGDISGINASVVNIQKEIDRLNEVAKNLNE
SLIDLQELGKYEQYIKWPWYIWLGFIAGLIAIVMVTIMLCCMTSCCSCLKGCCSCGSCCK
FDEDDSEPVLKGVKLHYT*

>BA.2 variant
MFVFLVLLPLVSSQCVNLITRTQSYTNSFTRGVYYPDKVFRSSVLHSTQDLFLPFFSNVT
WFHAIHVSGTNGTKRFDNPVLPFNDGVYFASTEKSNIIRGWIFGTTLDSKTQSLLIVNNA
TNVVIKVCEFQFCNDPFLDVYYHKNNKSWMESEFRVYSSANNCTFEYVSQPFLMDLEG
KQGNFKNLREFVFKNIDGYFKIYSKHTPINLGRDLPQGFSALEPLVDLPIGINITRFQTLLA
LHRSYLTPGDSSSGWTAGAAAYYVGYLQPRTFLLKYNENGTITDAVDCALDPLSETKCT
LKSFTVEKGIYQTSNFRVQPTESIVRFPNITNLCPFDEVFNATRFASVYAWNRKRISNCVA
DYSVLYNFAPFFAFKCYGVSPTKLNDLCFTNVYADSFVIRGNEVSQIAPGQTGNIADYN
YKLPDDFTGCVIAWNSNKLDSKVGGNYNYLYRLFRKSNLKPFERDISTEIYQAGNKPCN
GVAGFNCYFPLRSYGFRPTYGVGHQPYRVVVLSFELLHAPATVCGPKKSTNLVKNKCV
NFNFNGLTGTGVLTESNKKFLPFQQFGRDIADTTDAVRDPQTLEILDITPCSFGGVSVITP

GTNTSNQVAVLYQGVNCTEVPVAIHADQLTPTWRVYSTGSNVFQTRAGCLIGAEYVNN
SYECDIPIGAGICASYQTQTKSHRRARSVASQSIIAYTMSLGAENSVAYSNNSIAIPTNFTI
SVTTEILPVSMTKTSVDCTMYICGDSTECSNLLLQYGSFCTQLKRALTGIAVEQDKNTQE
VFAQVKQIYKTPPIKYFGGFNFSQILPDPSKPSKRSFIEDLLFNKVTLADAGFIKQYGDCL
GDIAARDLICAQKFNGLTVLPPLLTDEMIAQYTSALLAGTITSGWTFGAGAALQIPFAMQ
MAYRFNGIGVTQNVLYENQKLIANQFNSAIGKIQDSLSSTASALGKLQDVVNHNAQALN
TLVKQLSSKFGAISSVLNDILSRLDKVEAEVQIDRLITGRLQSLQTYVTQQLIRAAEIRAS
ANLAATKMSECVLGQSKRVDFCGKGYHLMSFPQSAPHGVVFLHVTYVPAQEKNFTTAP
AICHDGKAHFPREGVFVSNGTHWFVTQRNFYEPQIITTDNTFVSGNCDVVIGIVNNTVY
DPLQPELDSFKEELDKYFKNHTSPDVDLGDISGINASVVNIQKEIDRLNEVAKNLNESLID
LQELGKYEQYIKWPWYIWLGFIAGLIAIVMVTIMLCCMTSCCSCLKGCCSCGSCCKFDE
DDSEPVLKGVKLHYT*

Other virus strains. SARS pseudovirus, influenza A viruses (H1N1, H3N2, H7N9, H5N1, H10N8), chimpanzee adenovirus serotype 68 (AdC68), human adenovirus serotype 7 (Ad7), Middle East respiratory syndrome (MERS) pseudovirus, Marburg virus (MARV) pseudovirus, Ebola virus (EBOV) pseudovirus, vesicular stomatitis virus (VSV), and rabies challenge virus standard 11 (CVS-11) were obtained according to previously reported procedures [8].

Fluorescence quenching assay. Peptide probes (with a final concentration of 2 μM) were incubated with different concentrations of quenching materials including AuNPs (0-20 μg mL^{-1}), CNTs (0-8 μg mL^{-1}), 2D MnO_2 (0-12 μg mL^{-1}) and GO (0-15 μg mL^{-1}) in PBS (0.01 M, pH 7.4) for 2 min, and then the fluorescence was measured on a Varian Cary Eclipse fluorescence spectrophotometer with an excitation wavelength of 520 nm.

Fluorescent titration of peptide-material ensembles with SARS-Cov-2 pseudoviruses or recombinant RBDs. The fluorescent spectra of the peptide-material ensembles (2×10^{-6} M/15 μg mL^{-1}) with increasing viral titers were obtained in PBS (0.01 M, pH 7.40) with an excitation wavelength of 520 nm with the concentrations of SARS-Cov-2 pseudovirus (**WT**, B.1.1.7, B.1.351, P.1, B.1.617.2, or BA.2) and RBDs (**RBD-Alpha**, **RBD-Beta**, **RBD-Gamma**, **RBD-Omicron** and **RBD-Delta**) ranging from 0 to 5.0×10^{5} vp mL^{-1} and 0 to 12 μg mL^{-1}, respectively.

Differential sensing of RBDs with S_1-S_{20}. In a 384-well plate, quenching materials including AuNPs (10 uL, 60 μg mL^{-1}), CNTs (10 uL, 24 μg mL^{-1}), 2D MnO_2 (10 uL, 36 μg mL^{-1}), and GO (10 uL, 45 μg mL^{-1}) were first added, followed by addition of peptide probes (10 μL, 6 μM). The resulting mixtures were incubated for 5 minutes. Then, RBDs (10 μL, 20 μg mL^{-1}) were added, and the resulting mixtures were shaken at 37 °C for 15 minutes. The fluorescent intensity of each

well at 560 nm was recorded on an M5 Microplate reader with an excitation wavelength of 520 nm.

Differential sensing of SARS-Cov-2 pseudoviruses and other viruses with S_1-S_{20}. In a 384-well plate, quenching materials including AuNPs (10 uL, 60 μg mL^{-1}), CNTs (10 uL, 24 μg mL^{-1}), 2D MnO_2 (10 uL, 36 μg mL^{-1}), and GO (10 uL, 45 μg mL^{-1}) were added, followed by the addition of peptide probes (10 μL, 6 μM). The resulting mixtures were incubated for 5 minutes. Then, viruses (10 μL, 1.0×10^6 vp mL^{-1}) were added, and the resulting mixtures were shaken at 37°C for 15 minutes. The fluorescent intensity of each well at 560 nm was recorded on an M5 Microplate reader with an excitation wavelength of 520 nm.

S2. Additional figures

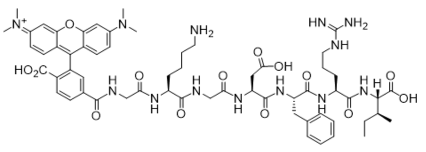

TI7 (TAMRA-GKGDFRI)

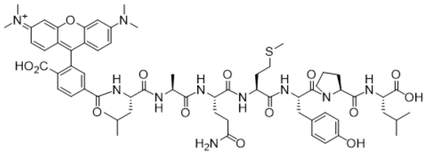

TL7 (TAMRA-LAQMYPL)

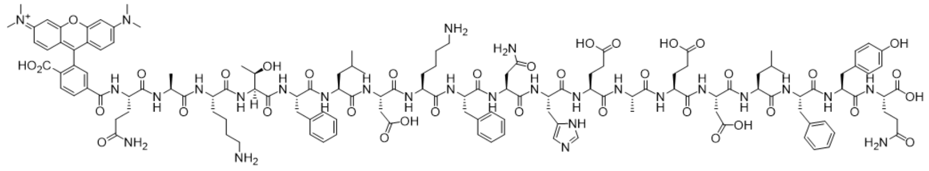

TQ19 (TAMRA-QAKTFLDKFNHEAEDLFYQ)

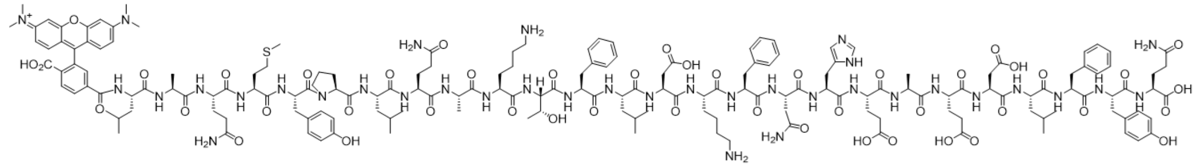

TQ26 (TAMRA-LAQMYPLQAKTFLDKFNHEAEDLFYQ)

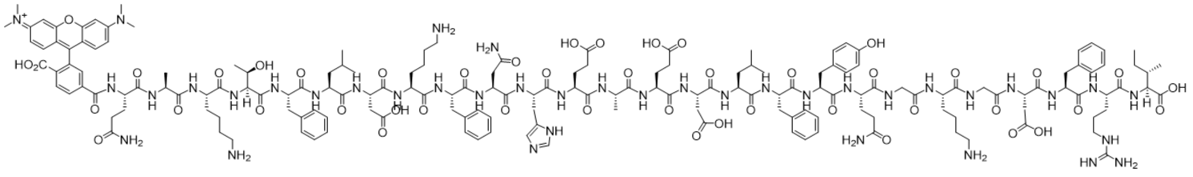

TI26 (TAMRA-QAKTFLDKFNHEAEDLFYQGKGDFRI)

Scheme S1. Structures of **TI7**, **TL7**, **TQ19**, **TQ26** and **TI26**.

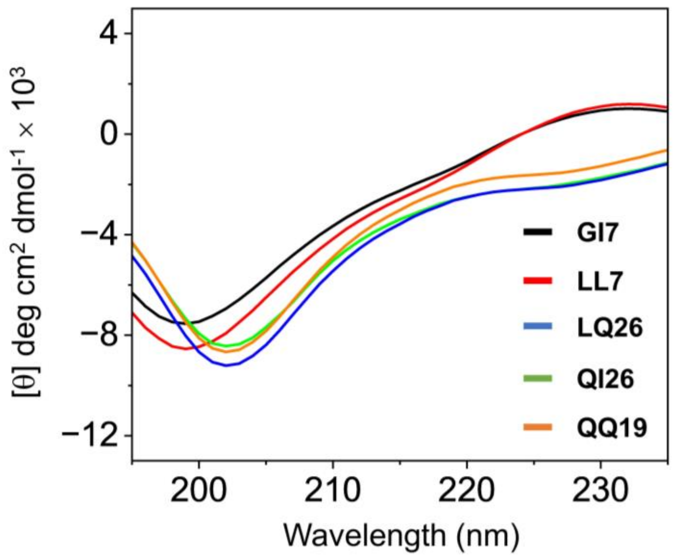

Figure S1. Circular dichroism spectra of **GI7**, **LL7**, **LQ26**, **QI26** and **QQ19** measured in 2 mM Tris-HCl (pH 8.0).

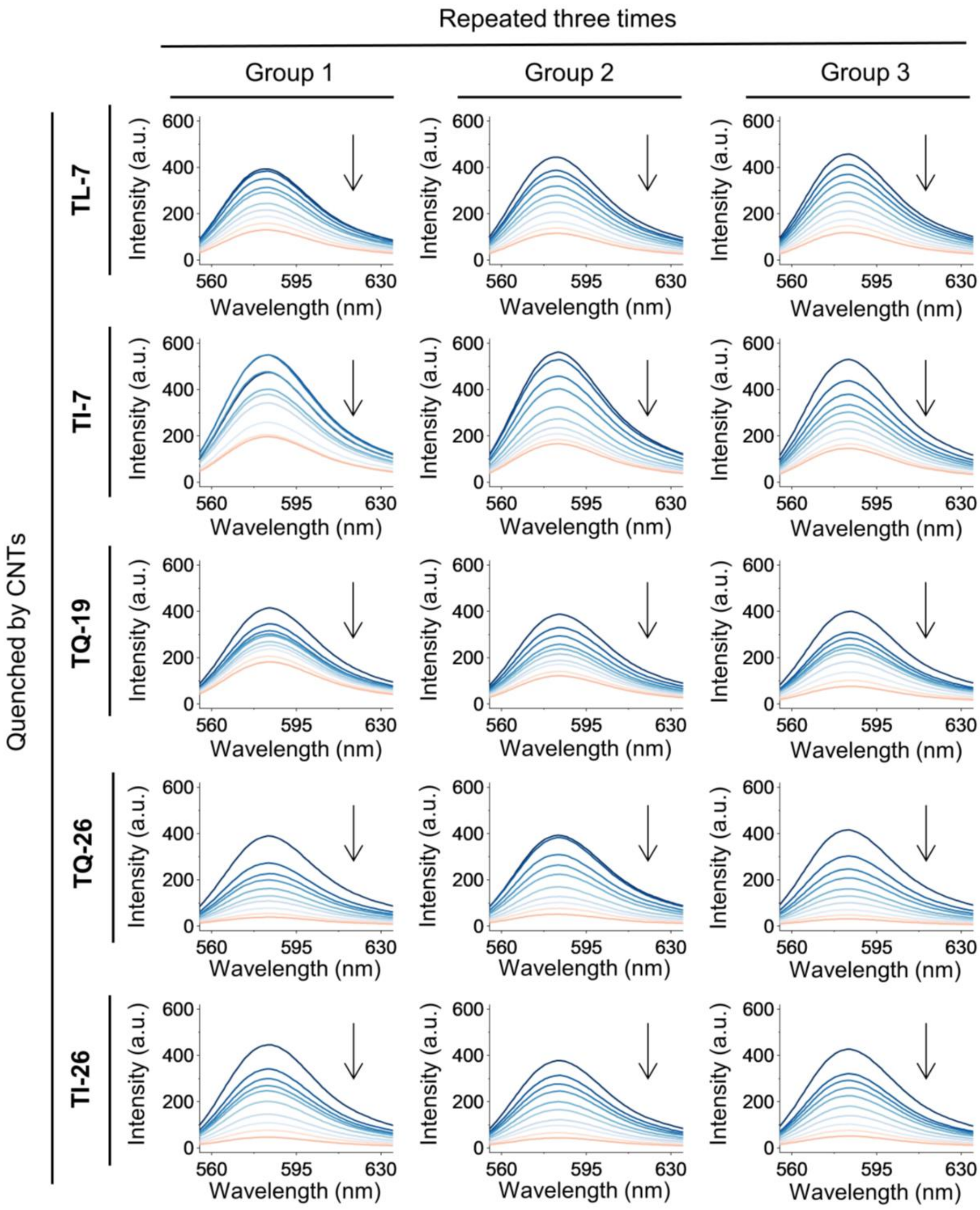

Figure S2. Fluorescence titration of peptide probes (2×10^{-6} M) in the presence of increasing CNTs (1–8 μg mL^{-1}) measured in PBS (0.01 M, pH 7.4); λ_{ex} = 520 nm.

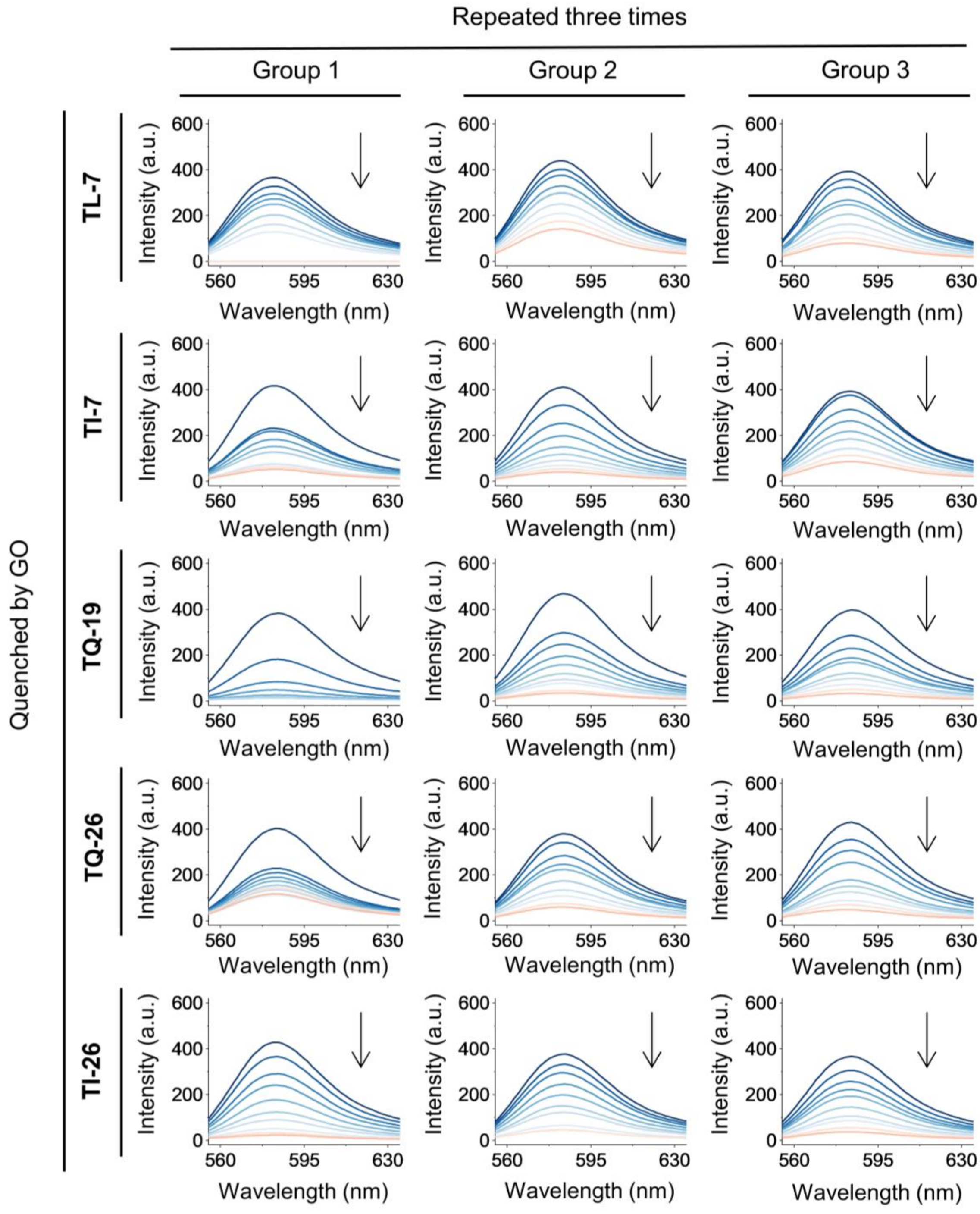

Figure S3. Fluorescence titration of peptide probes (2×10^{-6} M) in the presence of increasing GO (1–15 μg mL^{-1}) measured in PBS (0.01 M, pH 7.4); λ_{ex} = 520 nm.

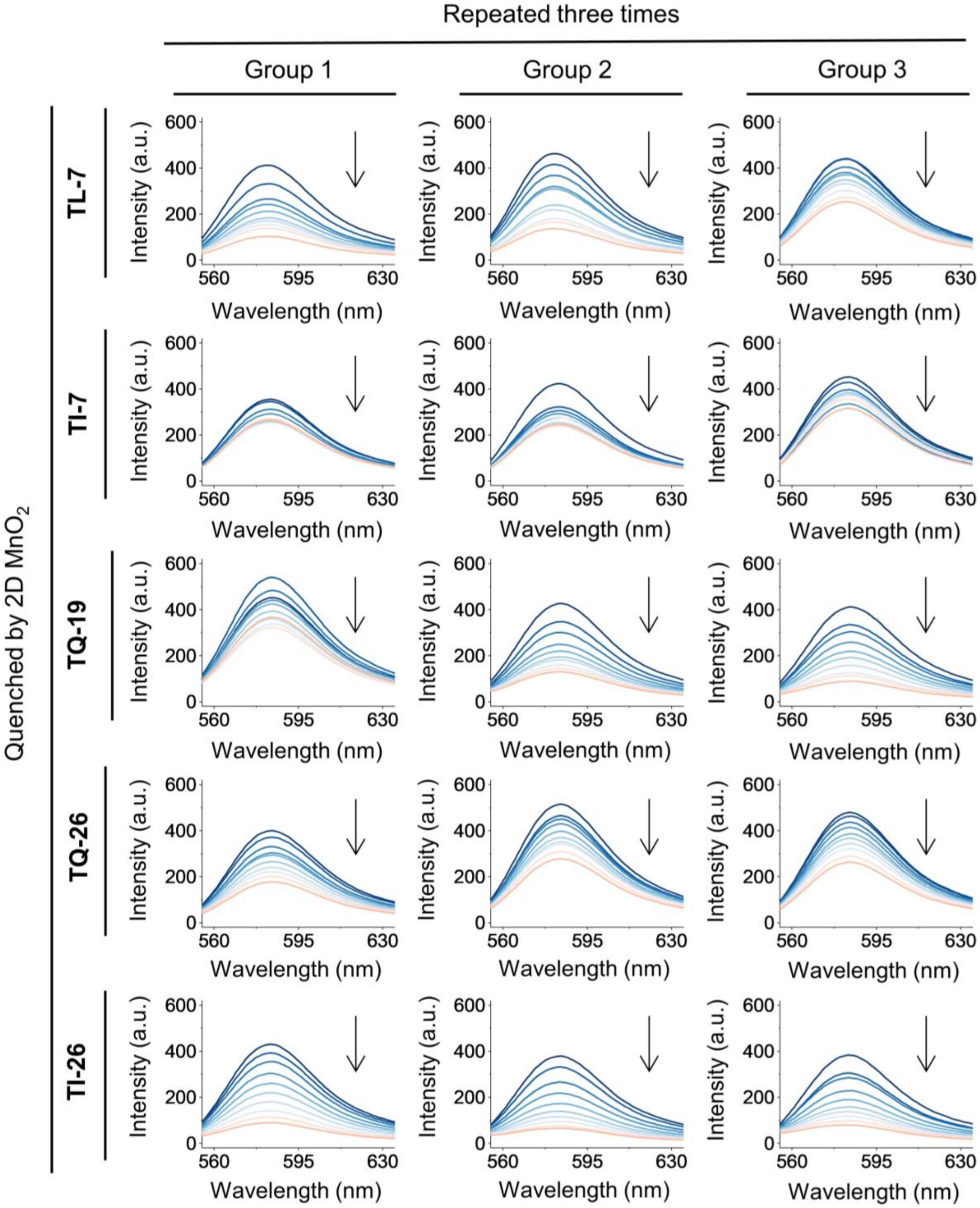

Figure S4. Fluorescence titration of peptide probes (2×10^{-6} M) in the presence of increasing 2D MnO_2 (1–12 μg mL^{-1}) measured in PBS (0.01 M, pH 7.4); λ_{ex} = 520 nm.

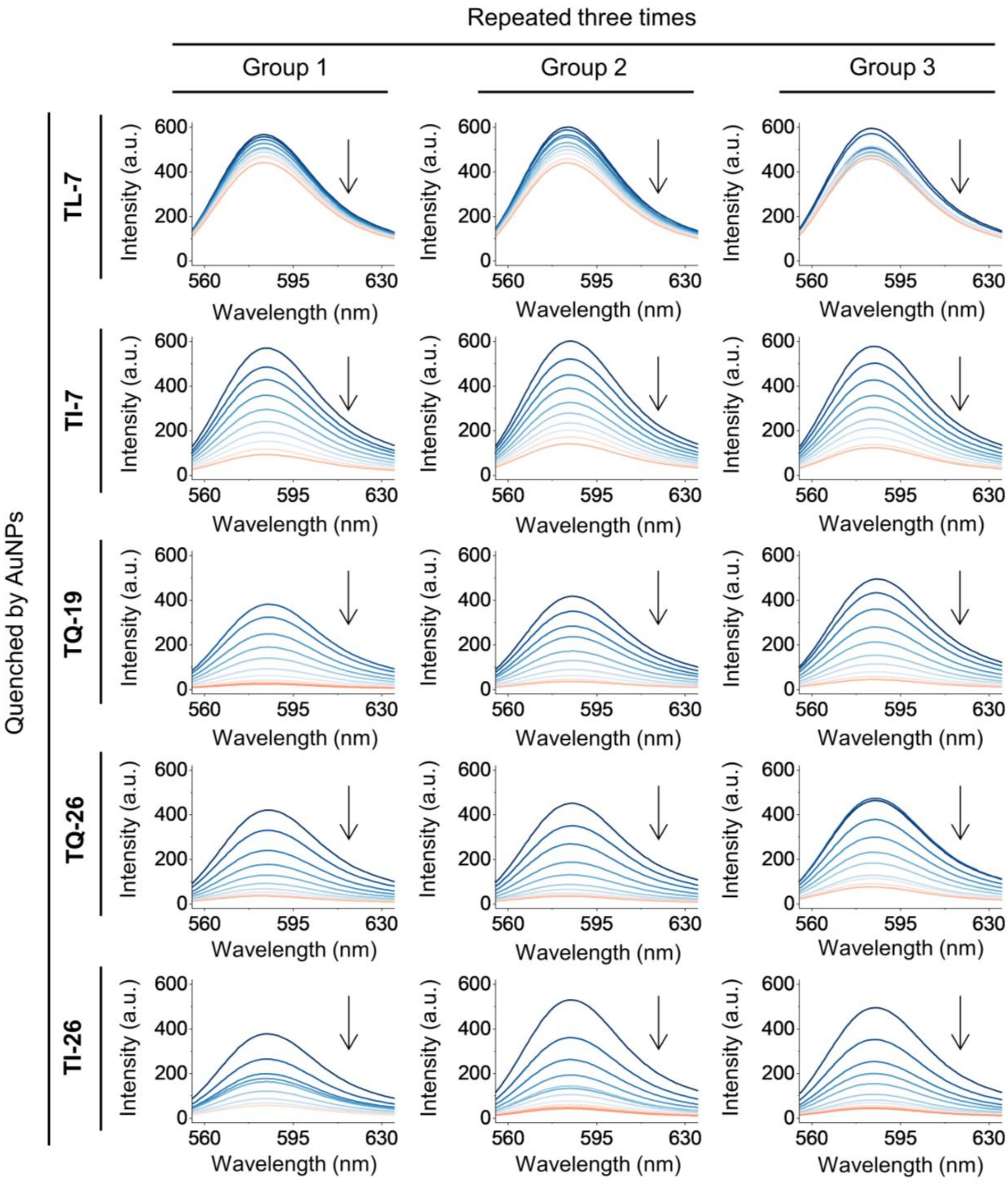

Figure S5. Fluorescence titration of peptide probes (2×10^{-6} M) in the presence of increasing AuNPs (1–20 μg mL^{-1}) measured in PBS (0.01 M, pH 7.4); λ_{ex} = 520 nm.

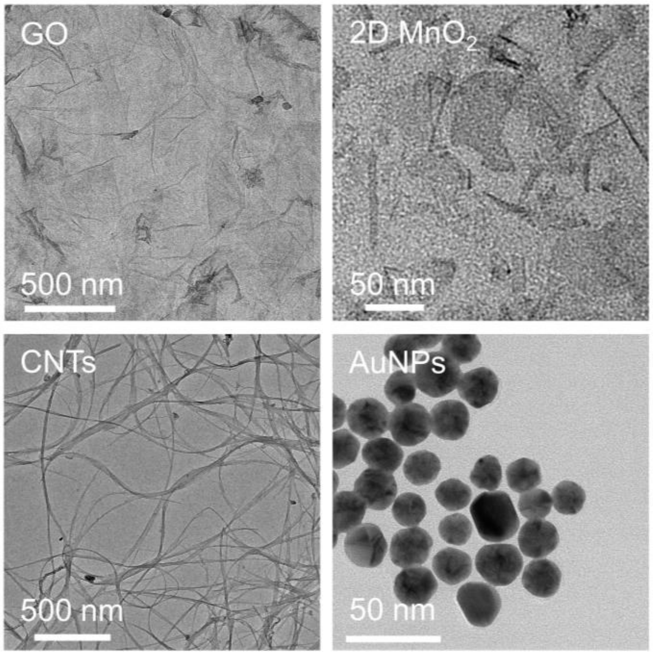

Figure S6. High-resolution transmission electron microscopic images of GO (2 μg mL^{-1}), 2D MnO_2 (2 μg mL^{-1}), CNTs (2 μg mL^{-1}) and AuNPs (2 μg mL^{-1}).

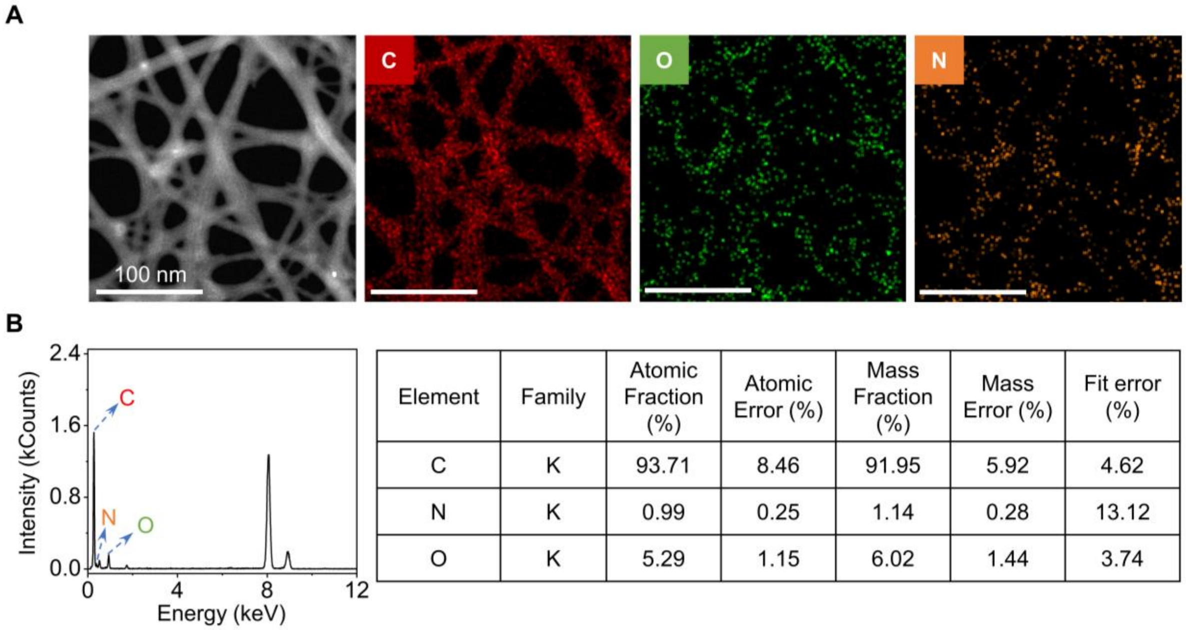

Element	Family	Atomic Fraction (%)	Atomic Error (%)	Mass Fraction (%)	Mass Error (%)	Fit error (%)
C	K	93.71	8.46	91.95	5.92	4.62
N	K	0.99	0.25	1.14	0.28	13.12
O	K	5.29	1.15	6.02	1.44	3.74

Figure S7. (A) Dark-field TEM image of **TI26**/CTNs and its corresponding EDX mapping images. (B) EDX spectrum and quantitative analysis of elemental distribution in **TI26**/CTNs.

A

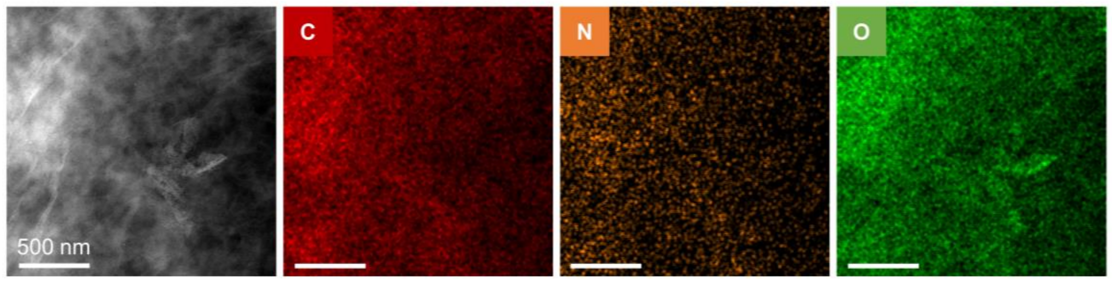

B

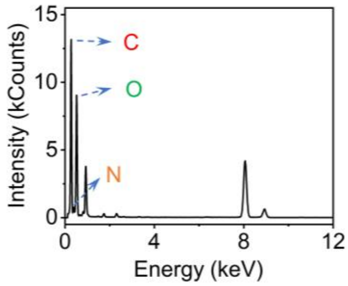

Element	Family	Atomic Fraction (%)	Atomic Error (%)	Mass Fraction (%)	Mass Error (%)	Fit error (%)
C	K	71.02	9.14	64.84	5.45	4.00
N	K	0.58	0.30	0.62	0.31	45.28
O	K	28.40	6.66	34.54	7.36	0.09

Figure S8. (A) Dark-field TEM image of **TI26**/GO and its corresponding EDX mapping images. (B) EDX spectrum and quantitative analysis of elemental distribution in **TI26**/GO.

A

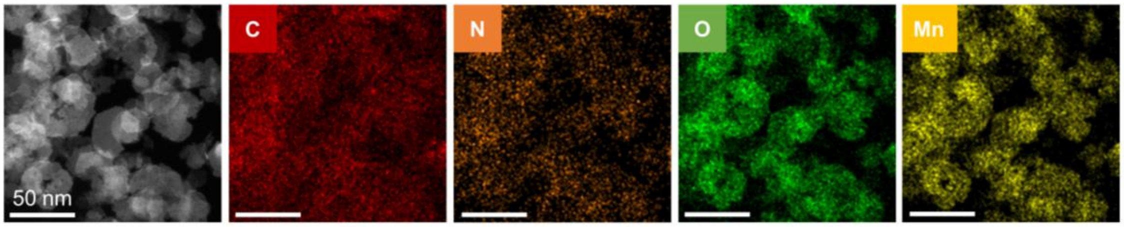

B

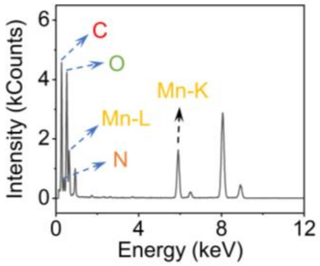

Element	Family	Atomic Fraction (%)	Atomic Error (%)	Mass Fraction (%)	Mass Error (%)	Fit error (%)
C	K	57.58	6.94	40.70	3.06	2.98
N	K	4.15	0.97	3.42	0.73	2.27
O	K	29.61	6.92	27.89	5.96	3.12
Mn	K	8.66	1.54	27.99	4,21	0.32

Figure S9. (A) Dark-field TEM image of **TI26**/2D MnO_2 and its corresponding EDX mapping images. (B) EDX spectrum and quantitative analysis of elemental distribution in **TI26**/2D MnO_2.

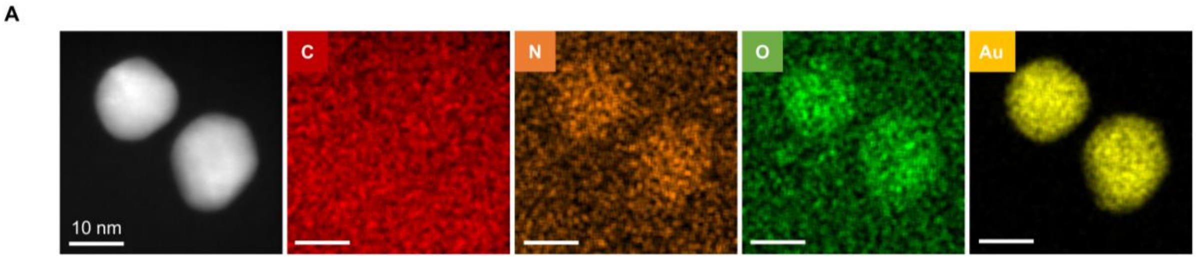

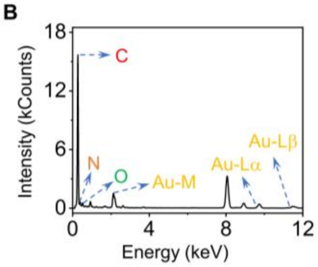

Element	Family	Atomic Fraction (%)	Atomic Error (%)	Mass Fraction (%)	Mass Error (%)	Fit error (%)
C	K	94.28	5.15	70.87	2.74	2.08
N	K	1.59	0.34	1.39	0.29	5.94
O	K	2.04	0.42	2.05	0.42	2.56
Au	K	2.08	0.26	25.69	2.99	0.46

Figure S10. (A) Dark-field TEM image of **TI26**/AuNPs and its corresponding EDX mapping images. (B) EDX spectrum and quantitative analysis of elemental distribution in **TI26**/AuNPs.

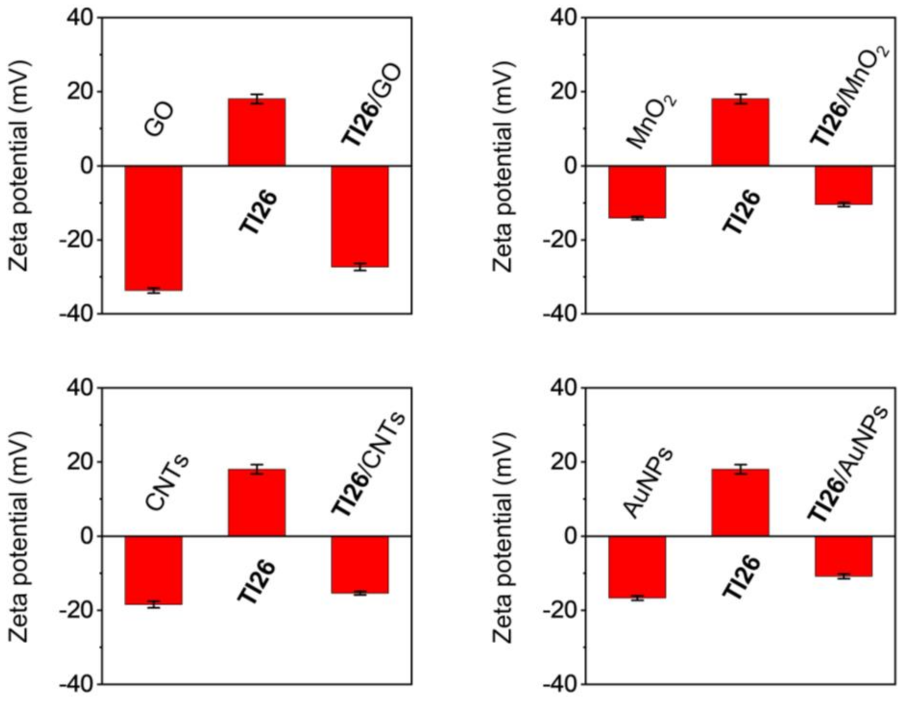

Figure S11. Zeta potential of peptide probes. Annotation: **TI26**/GO (1 μM/7.5 μg mL^{-1}), **TI26**/MnO_2 (1 μM/6 μg mL^{-1}), **TI26**/CNTs (1 μM/4 μg mL^{-1}), **TI26**/AuNPs (1 μM/10 μg mL^{-1}), GO (7.5 μg mL^{-1}), MnO_2 (6 μg mL^{-1}), CNTs (1 μM/4 μg mL^{-1}), AuNPs (1 μM/10 μg mL^{-1}) and **TI26** (1 μM).

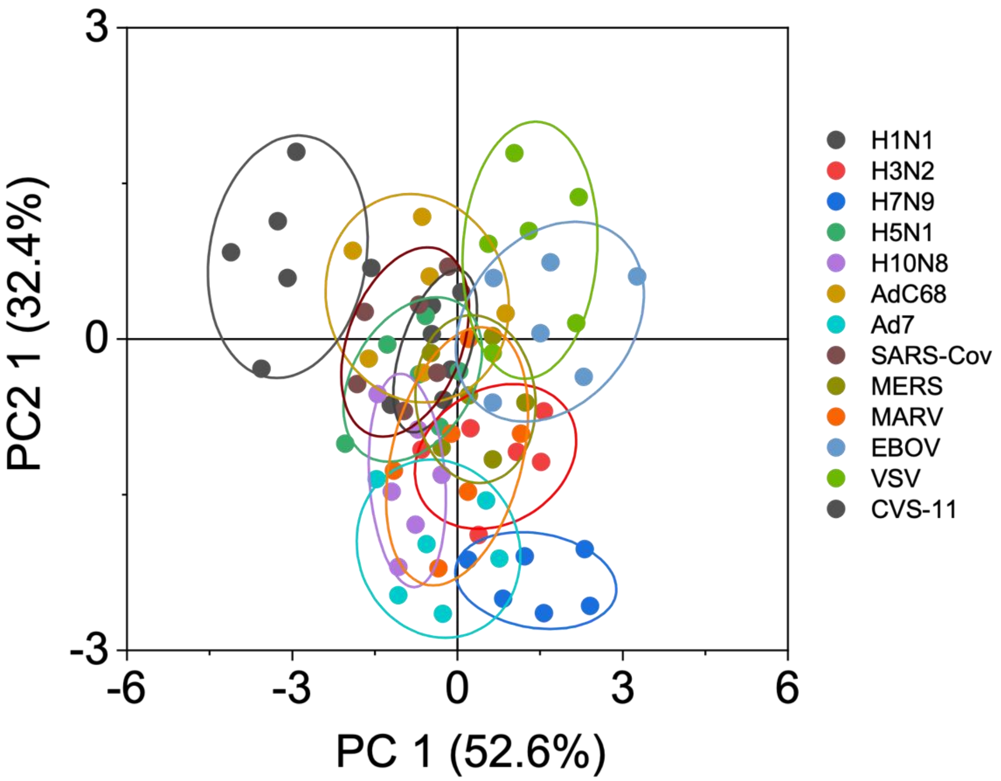

Figure S12. PCA of the fluorescence changes of the **S_1-S_{20}** sensor array to differentiate other viruses. All measurements were done on a M5 microplate reader in PBS (0.01 M, pH 7.4); λ_{em} = 560 nm, λ_{ex} = 520 nm.

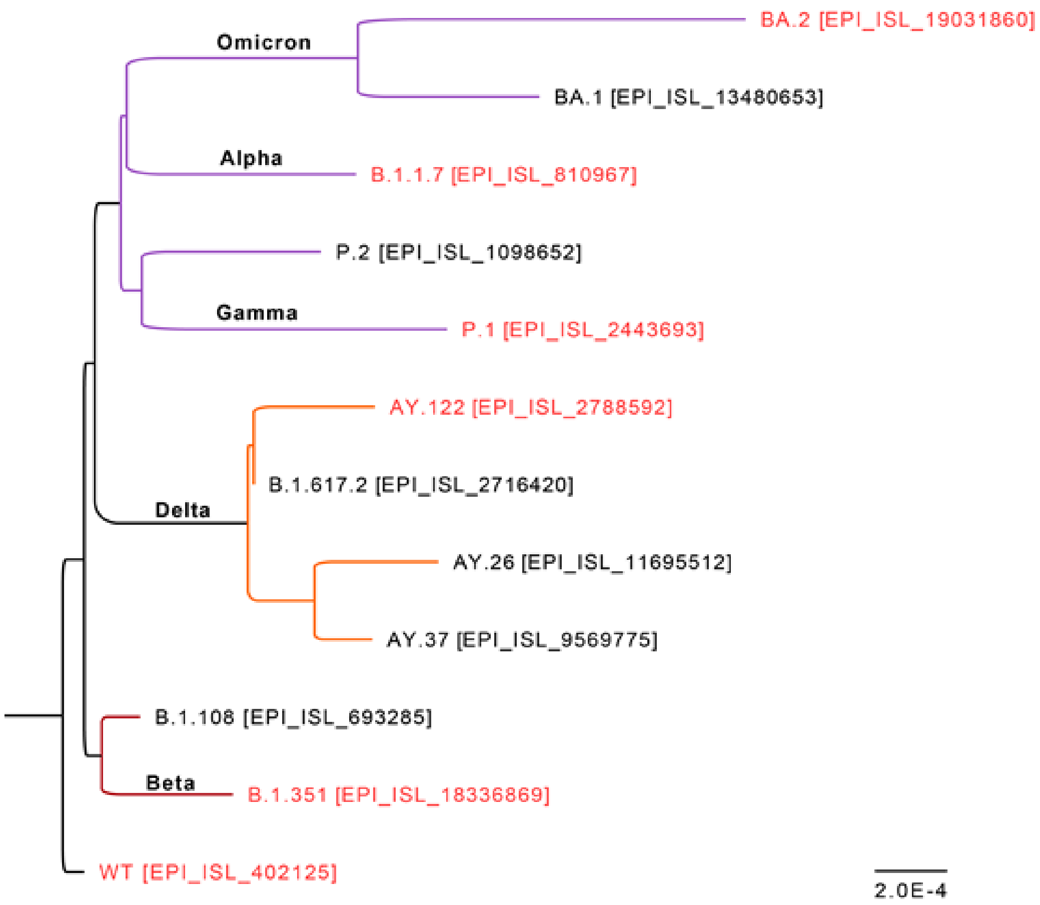

Figure S13. Phylogenetic tree of SARS-CoV-2 based on its complete genome sequence. The tree was constructed by the maximum likelihood method with 1000 bootstrap replicates using the IQ-TREE 2 software. Representative SARS-CoV-2 sequences from **WT** to **Omicron** were obtained from the GISAID database. The **WT** and VOCs, whose pseudoviruses were used for analyses in this study, are highlighted in red.

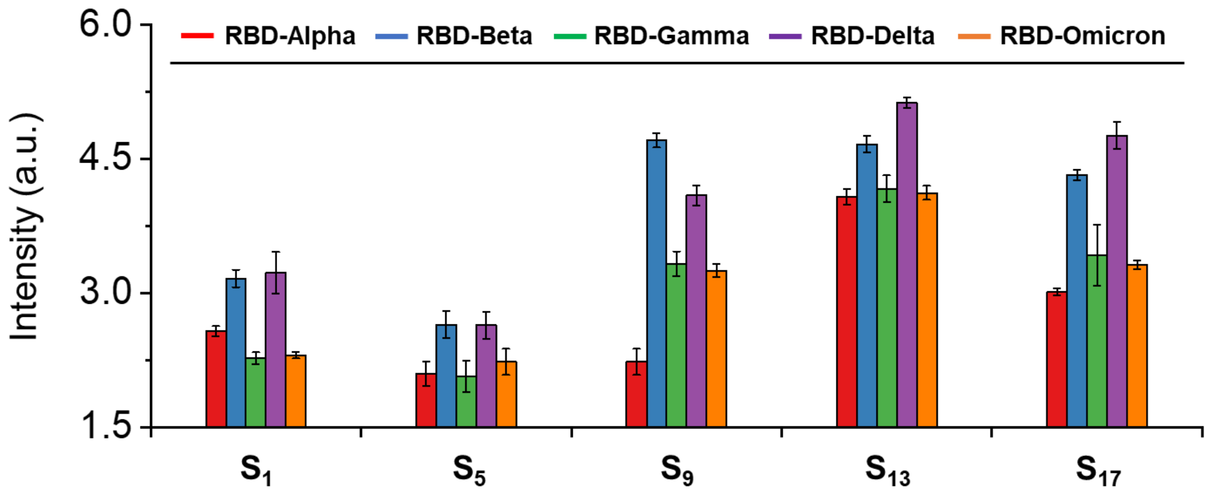

Figure S14. Fluorescence intensity changes of **S_1**, **S_5**, **S_9**, **S_{13}**, **S_{17}** seen in the presence of VOC RBDs. All measurements were done on a M5 microplate reader in PBS (0.01 M, pH 7.4); λ_{em} = 560 nm, λ_{ex} = 520 nm.

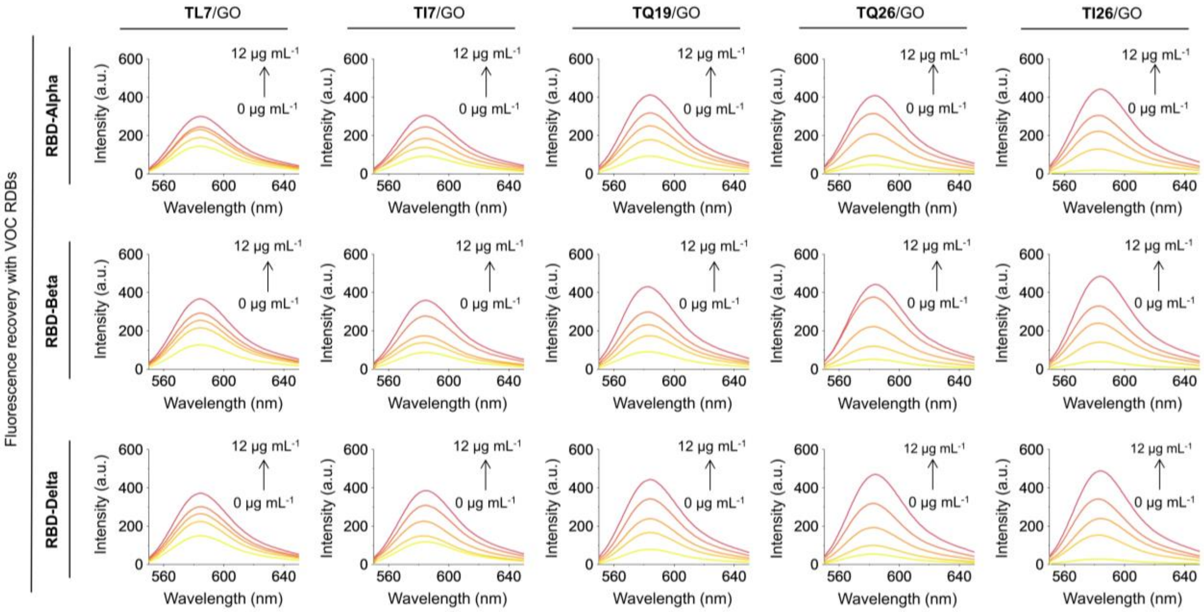

Figure S15. Fluorescence titration of peptide-GO ensembles (2×10^{-6} M/15 μg mL^{-1}) in the presence of increasing **RBD-Alpha**, **RBD-Beta** or **RBD-Delta**. The RBDs concentrations used range 0 to 12 μg mL^{-1}.

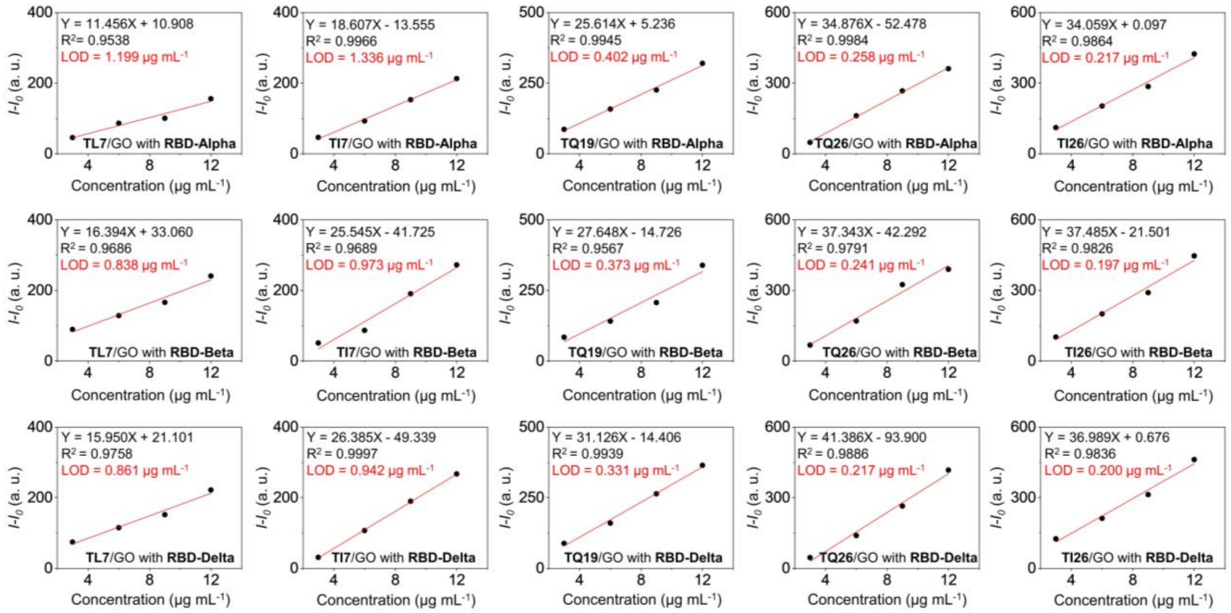

Figure S16. Plotting the fluorescent changes of peptide-GO ensembles in PBS (0.01 M, pH 7.4) as a function of VOC RBDs concentration. These data were used to determine detection limits (3σ/k).

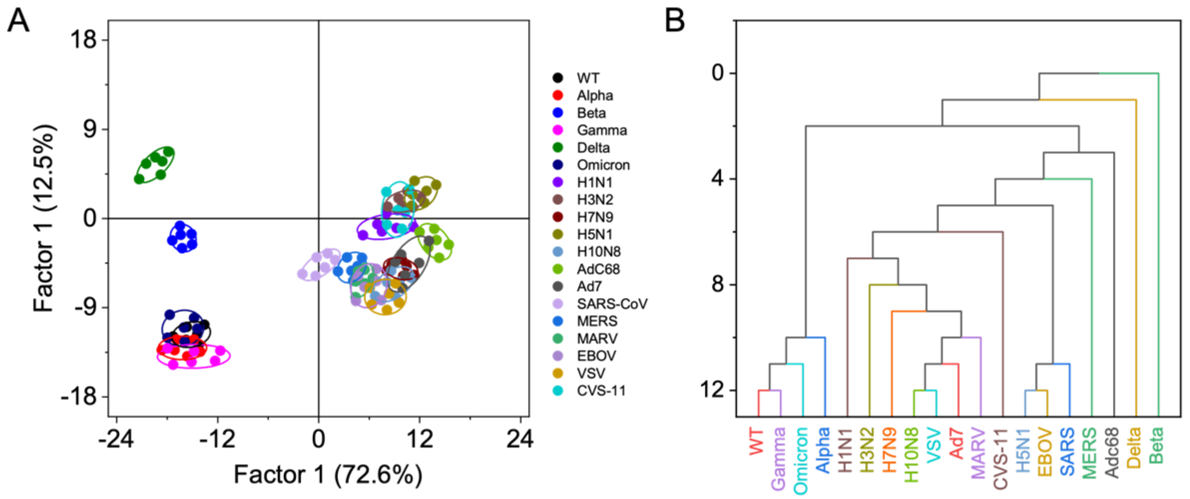

Figure S17. (A) LDA plot and (b) HCA dendrogram of the fluorescence changes of the **S_1-S_{20}** sensor array to differentiate SARS-CoV-2 from other viruses. The viruses used are #1: **WT**, #2: **Alpha**, #3: **Beta**, #4: **Gamma**, #5: **Delta**, #6: **Omicron**, #7: SARS-CoV, #8: H1N1, #9: H3N2, #10: H7N9, #11: H5N1, #12: H10N8, #13: MERS, #14: MARV, #15: AdC68, #16: Ad7, #17: EBOV, #18: VSV, and #19: CVC-11. All measurements were done on a M5 microplate reader in PBS (0.01 M, pH 7.4); λ_{em} = 560 nm, λ_{ex} = 520 nm.

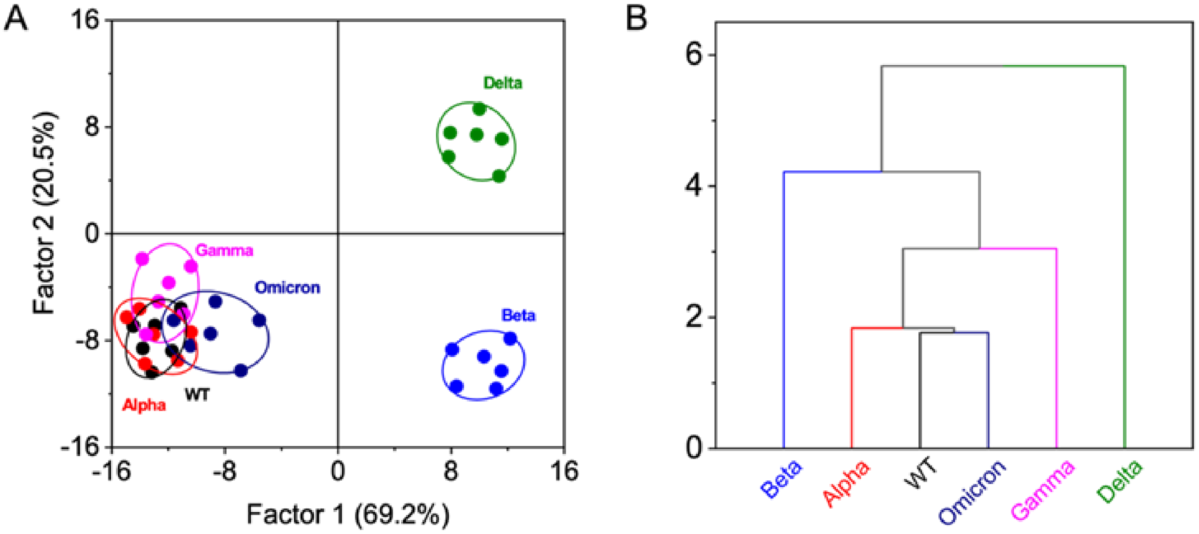

Figure S18. (A) LDA plot and (b) HCA dendrogram of the fluorescence changes of the **S1-S20** sensor array to classify VOCs of SARS-CoV-2. All measurements were done on a M5 microplate reader in PBS (0.01 M, pH 7.4); λ_{em} = 560 nm, λ_{ex} = 520 nm.

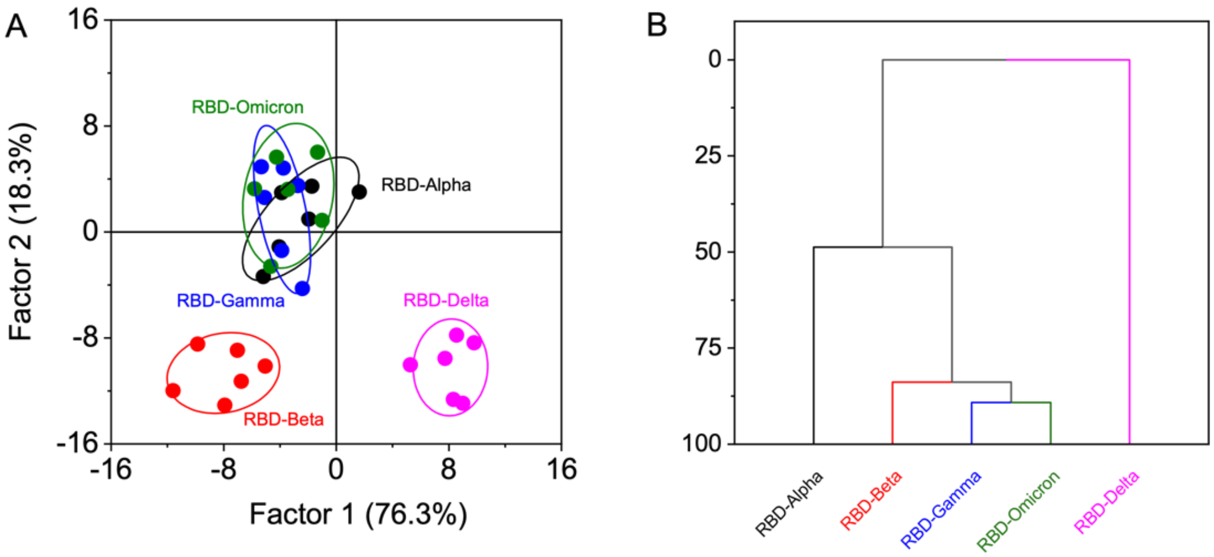

Figure S19. (A) LDA plot and (b) HCA dendrogram of the fluorescence changes of the **S_1-S_{20}** sensor array to classify RBDs from the VOCs. All measurements were done on a M5 microplate reader in PBS (0.01 M, pH 7.4); λ_{em} = 560 nm, λ_{ex} = 520 nm.

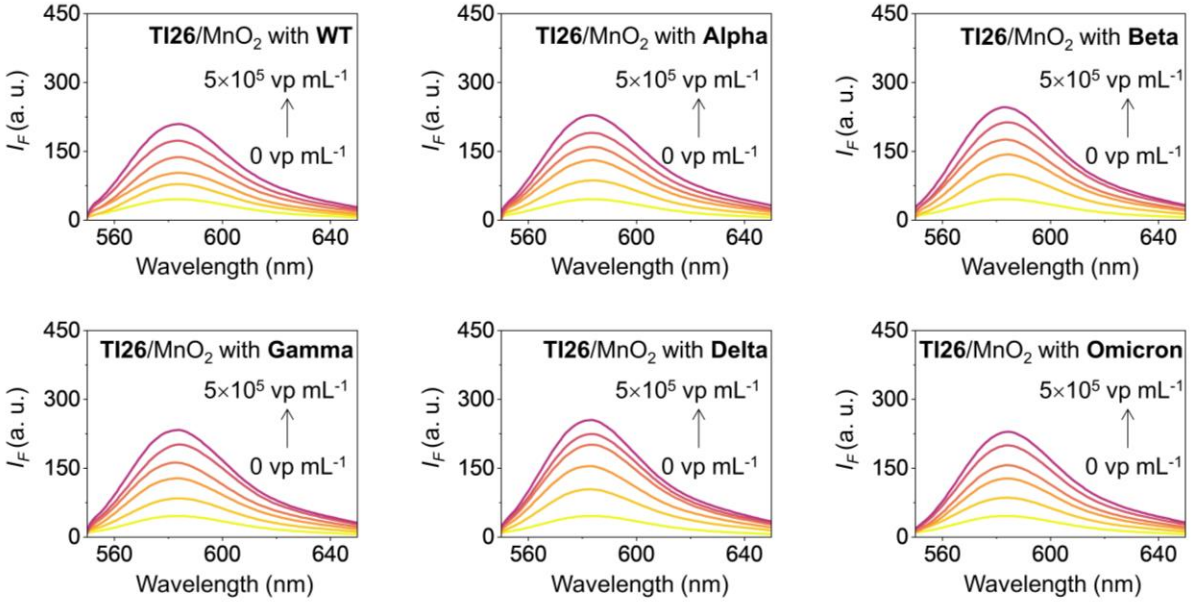

Figure S20. Fluorescence titration of **TI26**/2D MnO_2 (2×10^{-6} M/12 µg mL^{-1}) in the presence of increasing **WT**, **Alpha**, **Beta**, **Gamma**, **Delta** and **Omicron**. The virus concentrations used range 0 to 5.0×10^{5} vp mL^{-1}.

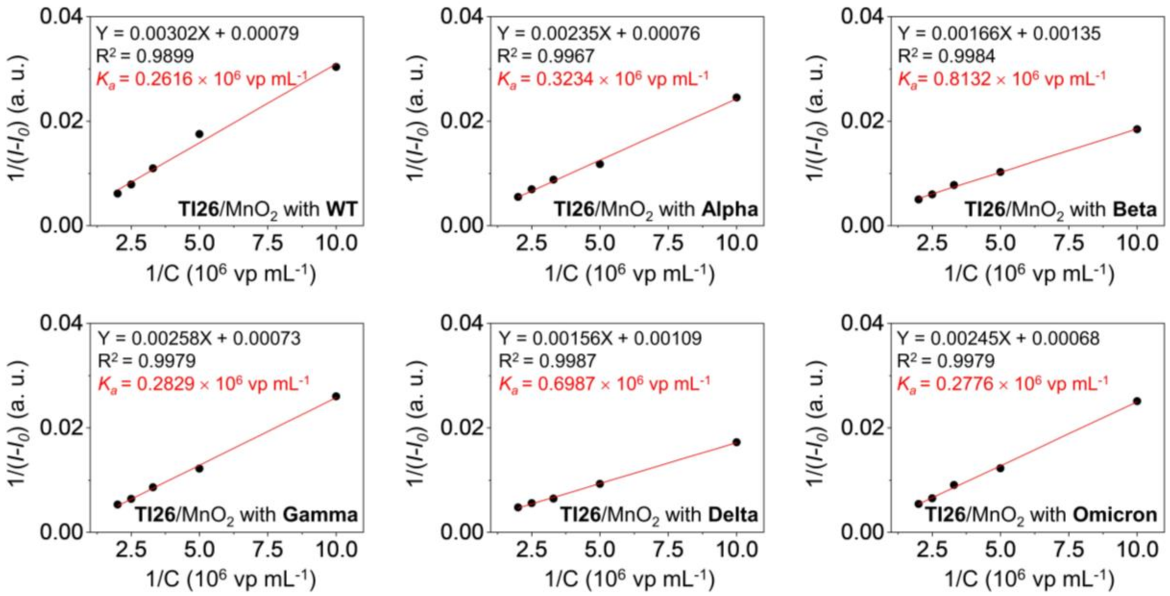

Figure S21. Double reciprocal linear fitting equation determining binding constant (K_a) from fluorescence titration of **TI26**/2D MnO_2 (2×10^{-6} M/12 µg mL^{-1}) in the presence of increasing **WT**, **Alpha**, **Beta**, **Gamma**, **Delta** and **Omicron** at the indicated concentrations.

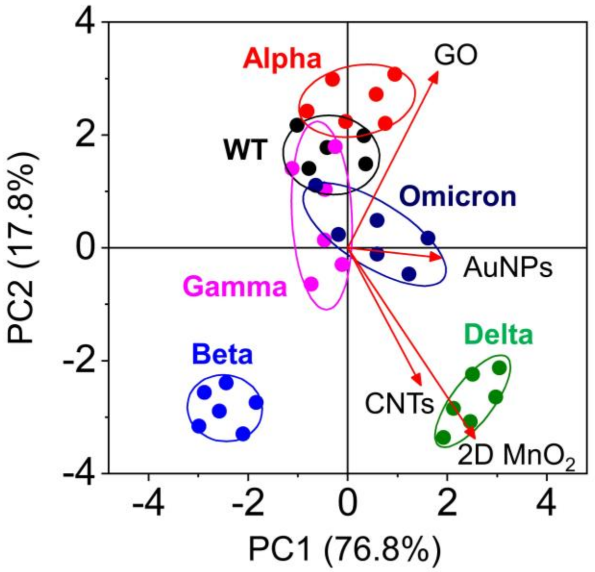

Figure S22. PCA and loading plots of the fluorescence changes of $\mathbf{S_1}$-$\mathbf{S_{20}}$ sensor array for VOCs. All measurements were done using a M5 microplate reader in PBS (0.01 M, pH 7.4); where λ_{em} = 560 nm; λ_{ex} = 520 nm.

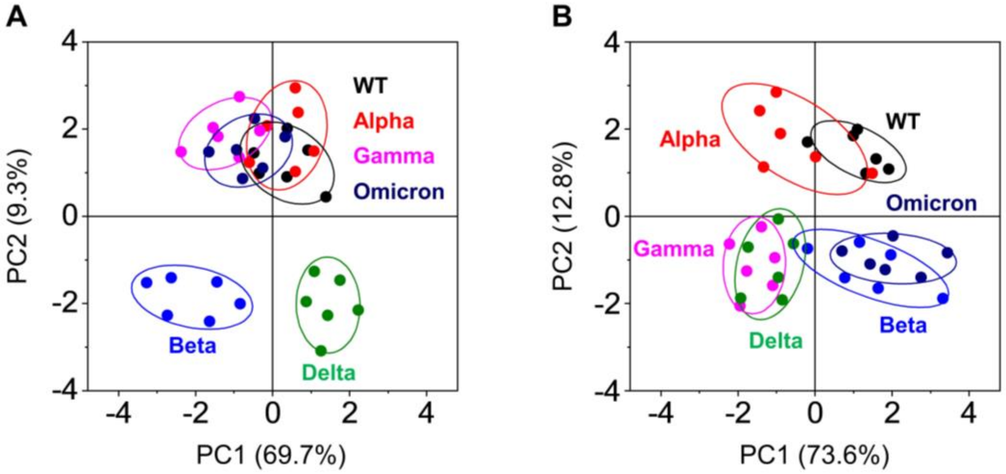

Figure S23. (A) PCA of the fluorescence changes of **S_1, S_2, S_4, S_5, S_6, S_8, S_9, S_{10}, S_{12}, S_{13}, S_{14}, S_{16}, S_{17}, S_{18}, S_{20}** sensor array for VOCs. (B) PCA of the fluorescence changes of **S_2, S_4, S_6, S_8, S_{10}, S_{12}, S_{14}, S_{16}, S_{18}, S_{20}** sensor array for VOCs. All measurements were done on a M5 microplate reader in PBS (0.01 M, pH 7.4); λ_{em} = 560 nm; λ_{ex} = 520 nm.

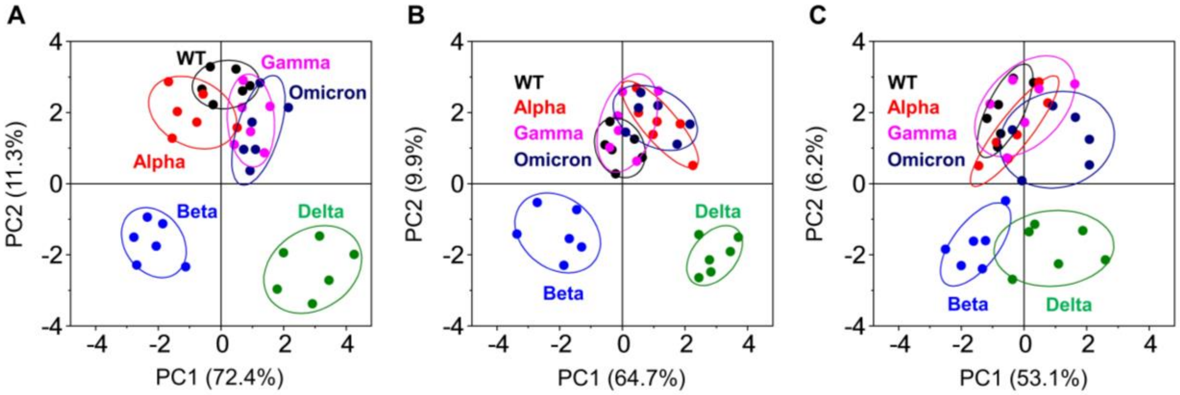

Figure S24. (A) PCA of the fluorescence changes of **S_5, S_6, S_7, S_8, S_9, S_{10}, S_{11}, S_{12}, S_{13}, S_{14}, S_{15}, S_{16}, S_{17}, S_{18}, S_{19}, S_{20}** sensor array for VOCs. (B) PCA of the fluorescence changes of **S_9, S_{10}, S_{11}, S_{12}, S_{13}, S_{14}, S_{15}, S_{16}, S_{17}, S_{18}, S_{19}, S_{20}** sensor array for VOCs. (C) PCA of the fluorescence changes of **S_{13}, S_{14}, S_{15}, S_{16}, S_{17}, S_{18}, S_{19}, S_{20}** sensor array for VOCs. All measurements were done on a M5 microplate reader in PBS (0.01 M, pH 7.4); λ_{em} = 560 nm; λ_{ex} = 520 nm.

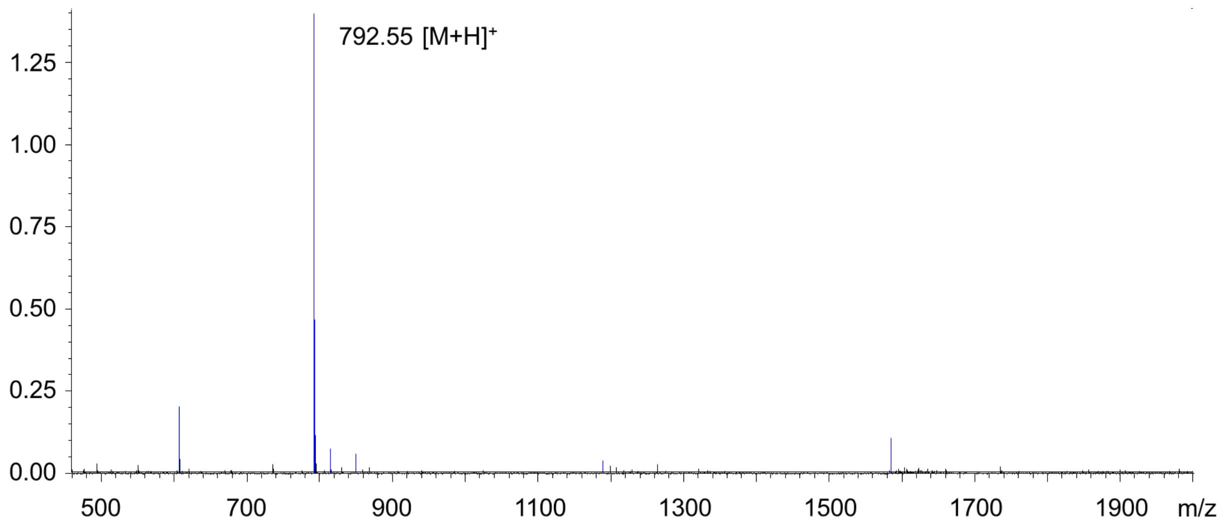

Figure S25. Mass spectrum of **GI-7**.

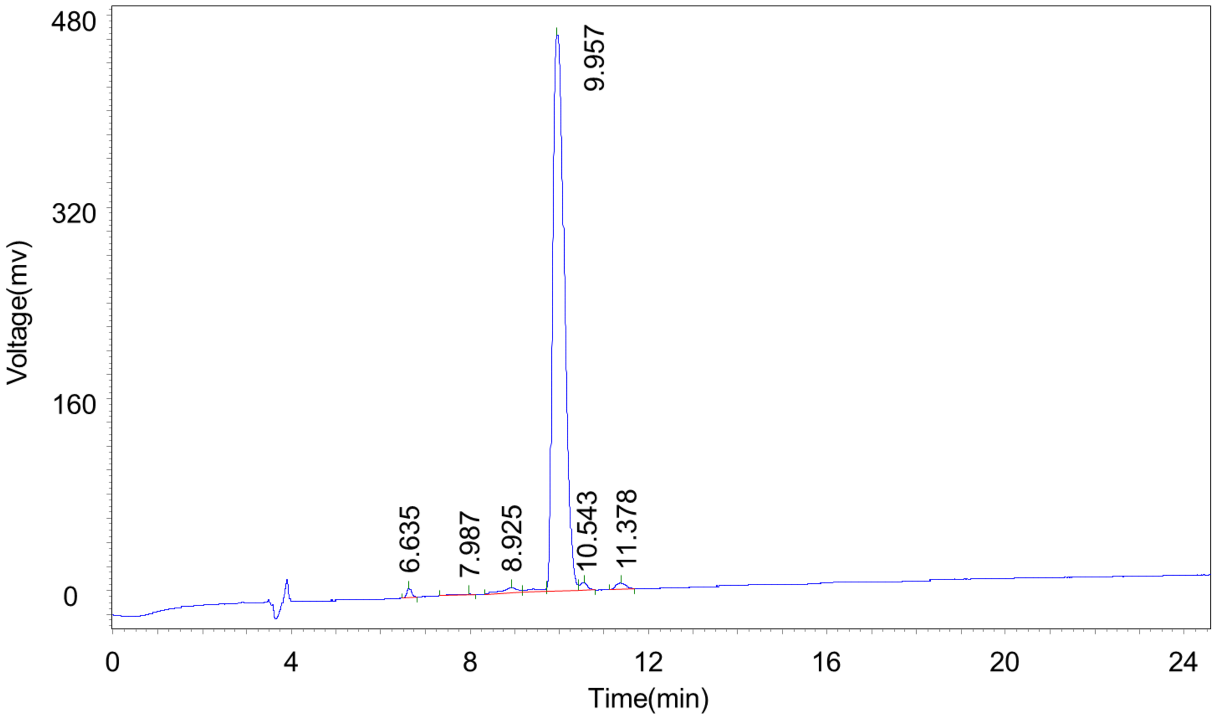

Figure S26. HPLC trace of **GI-7**.

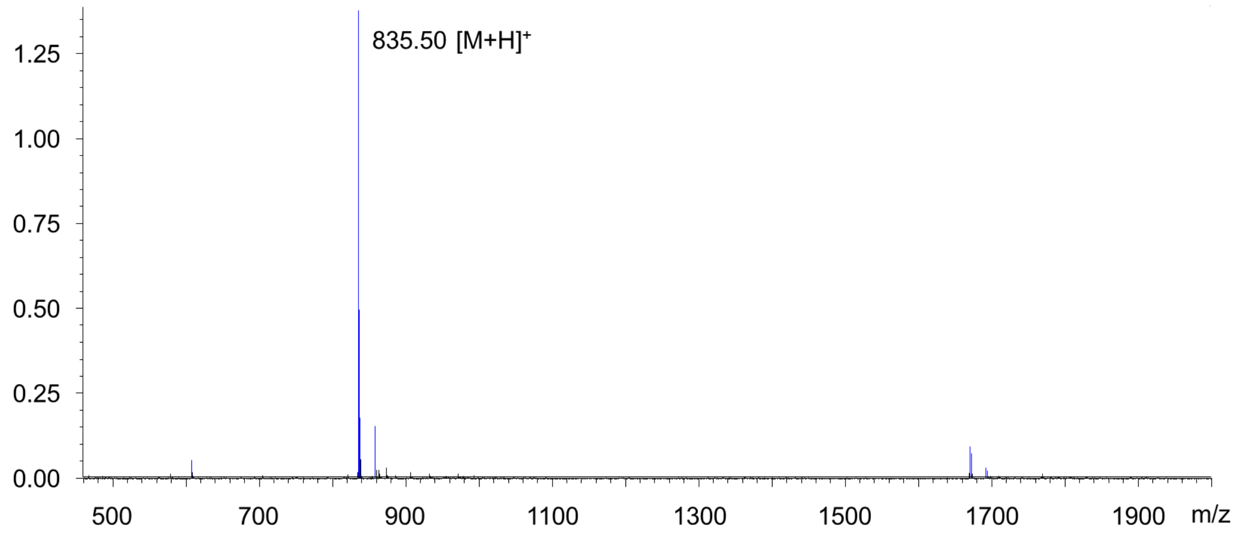

Figure S27. Mass spectrum of **LL-7**.

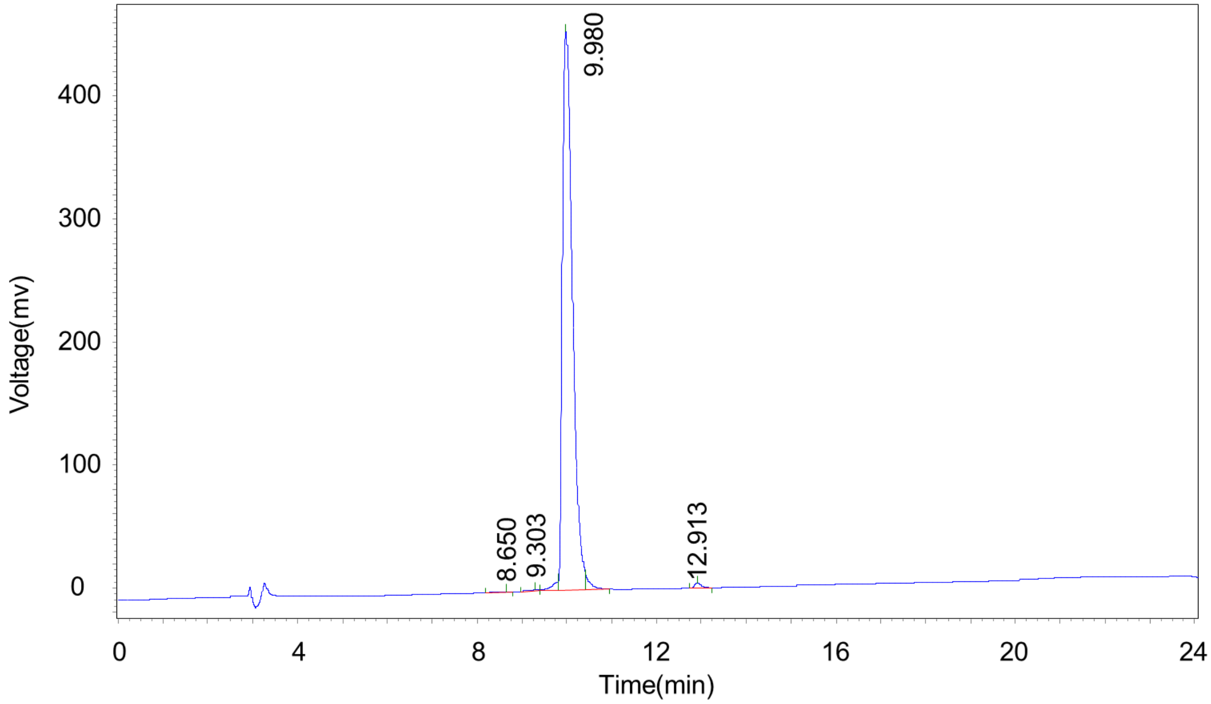

Figure S28. HPLC trace of **LL-7**.

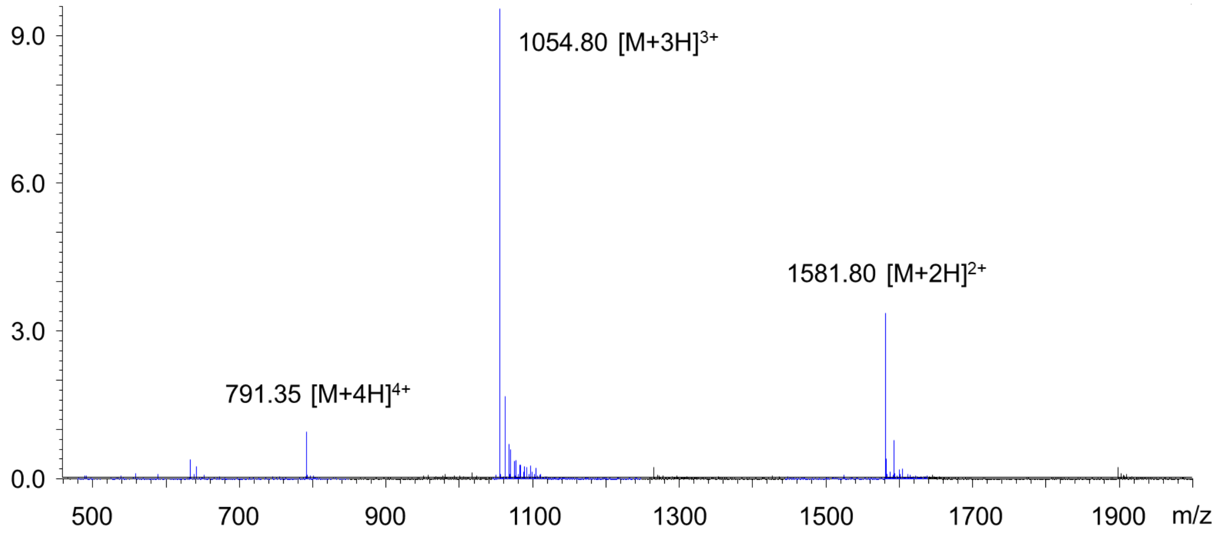

Figure S29. Mass spectrum of **LQ-26**.

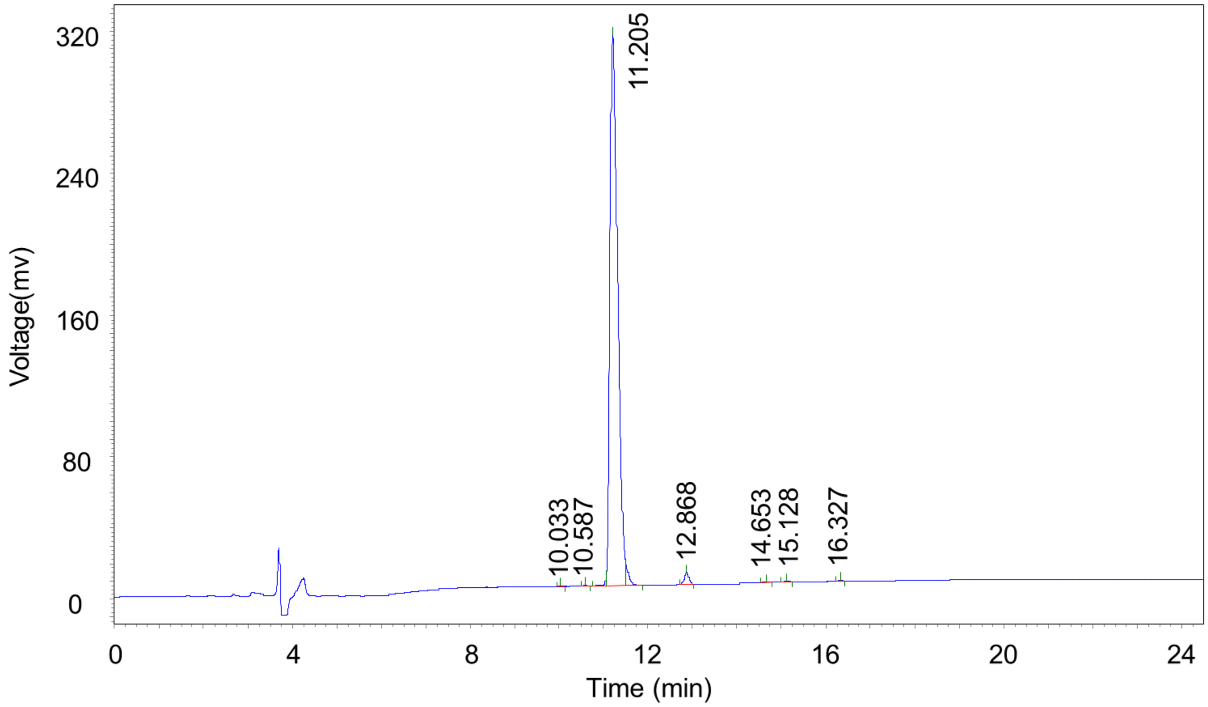

Figure S30. HPLC trace of **LQ-26**.

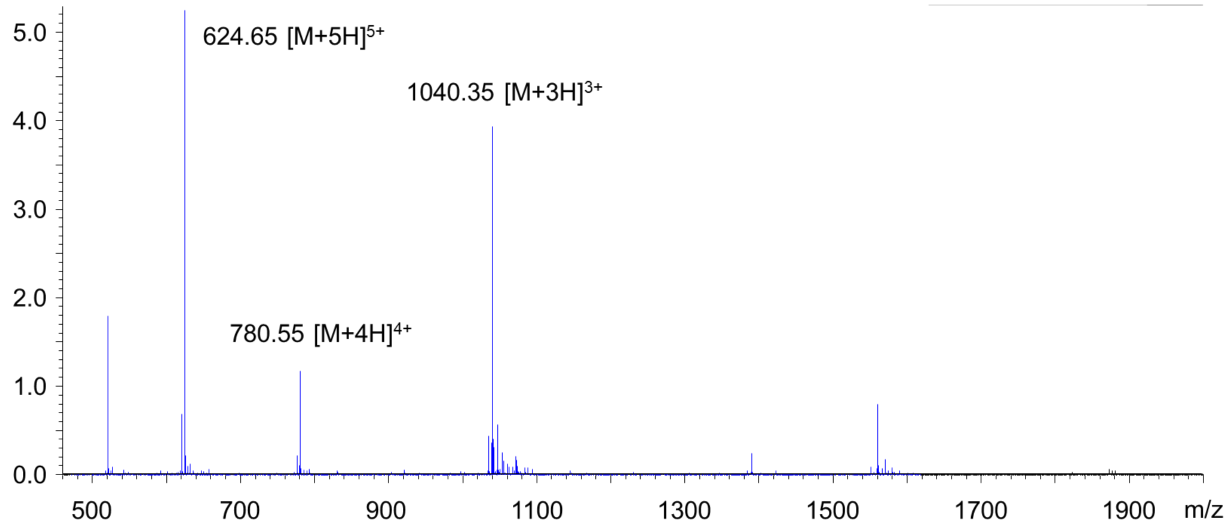

Figure S31. Mass spectrum of **QI-26**.

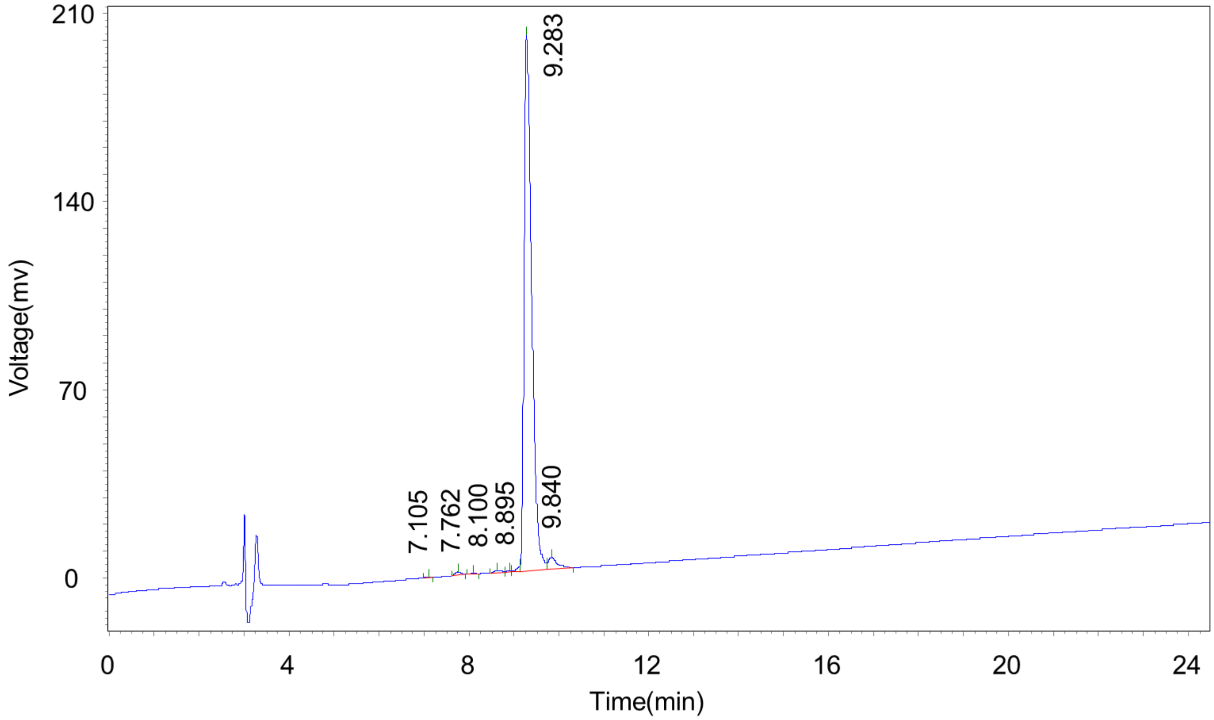

Figure S32. HPLC trace of **QI-26**.

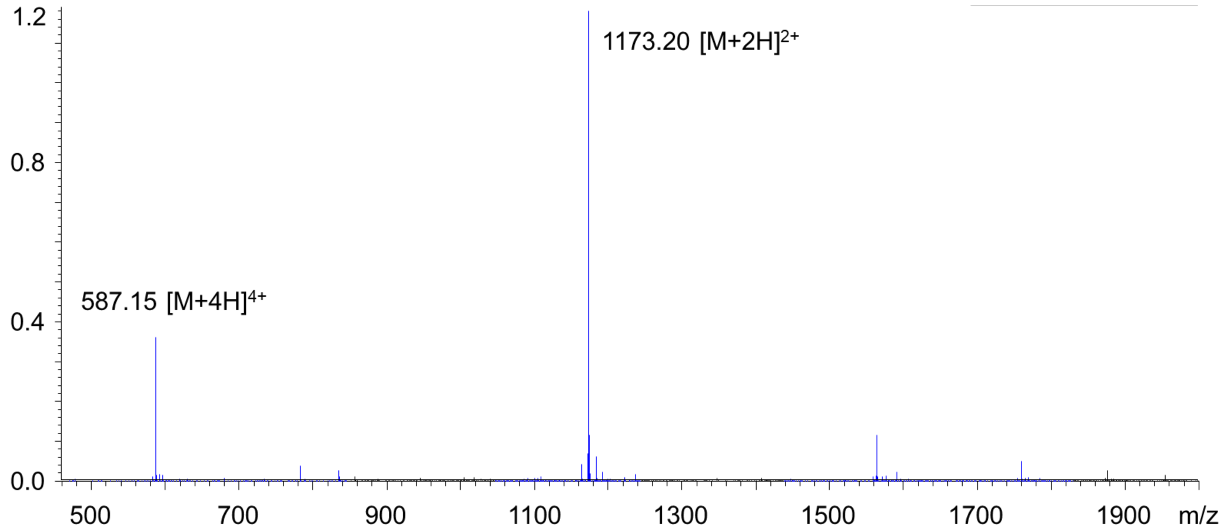

Figure S33. Mass spectrum of **QQ-19**.

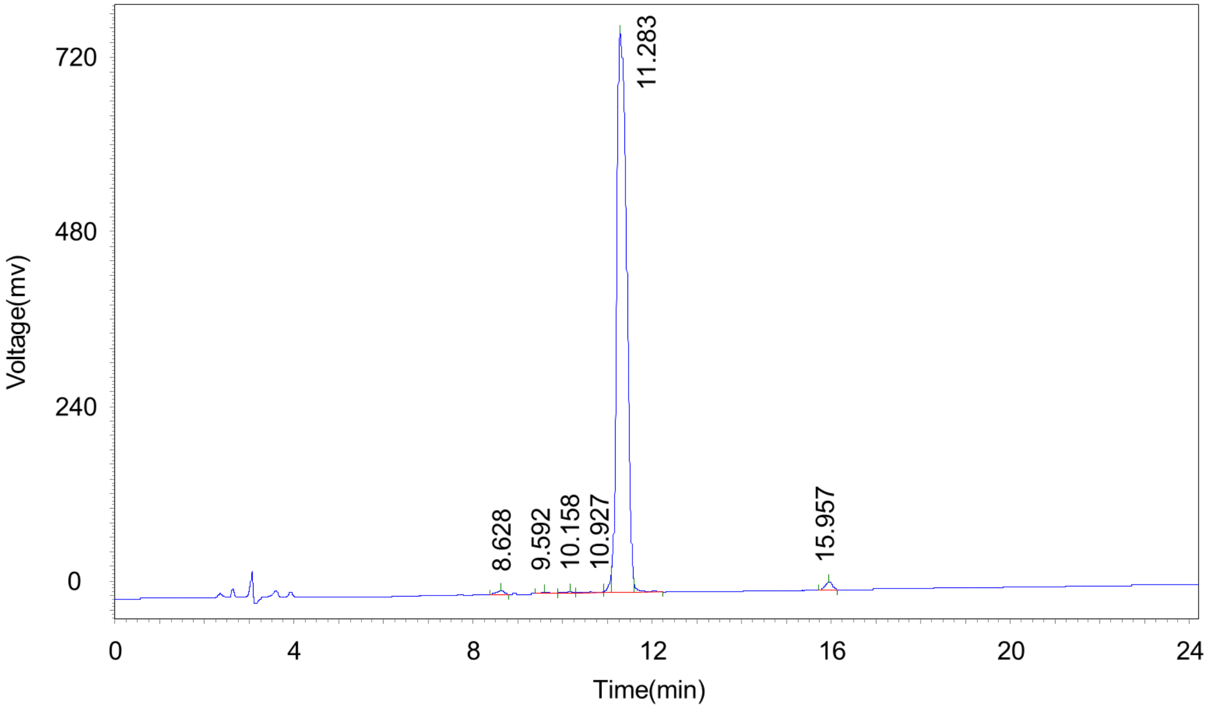

Figure S34. HPLC trace of **QQ-19**.

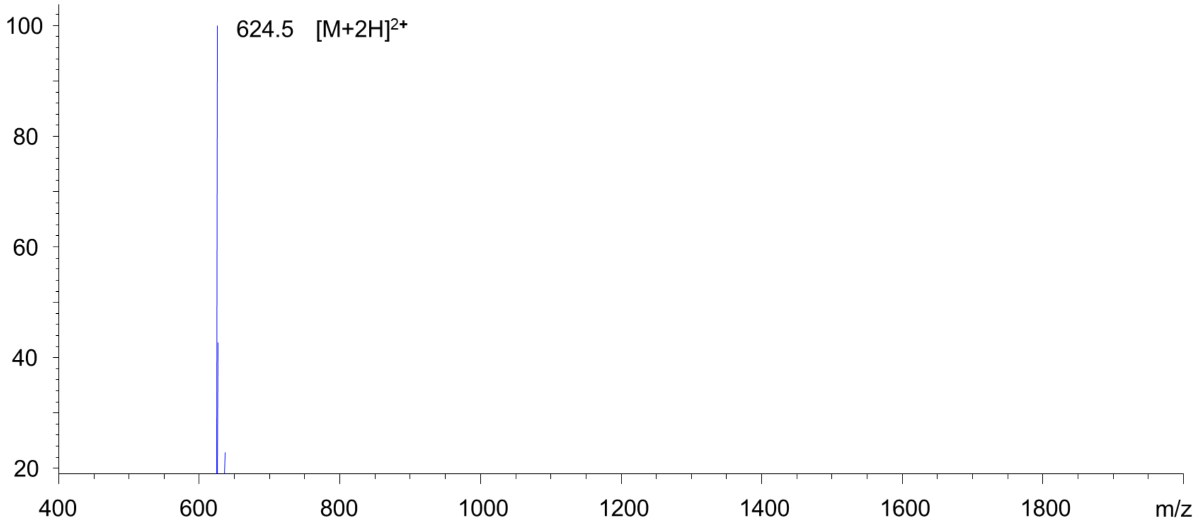

Figure S35. Mass spectrum of **TL-7**.

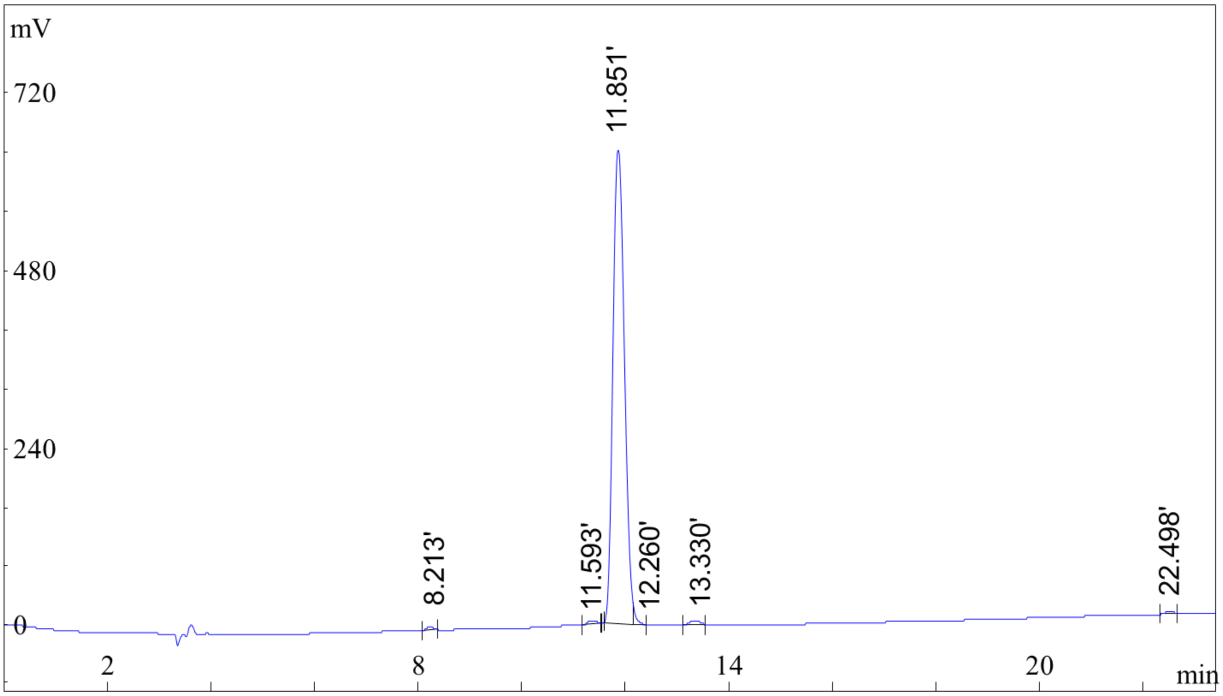

Figure S36. HPLC trace of **TL-7**.

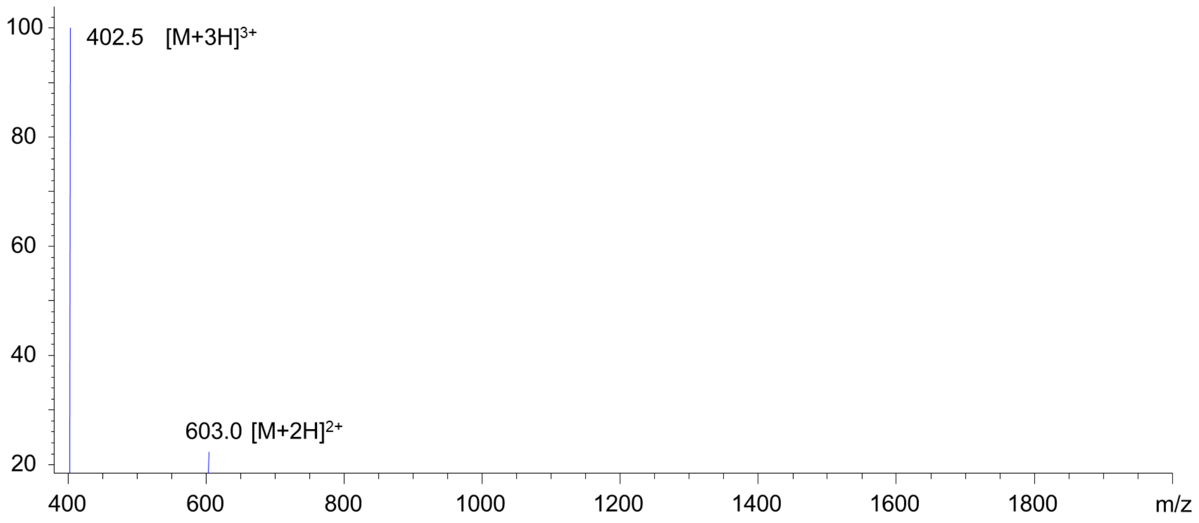

Figure S37. Mass spectrum of **TI-7**.

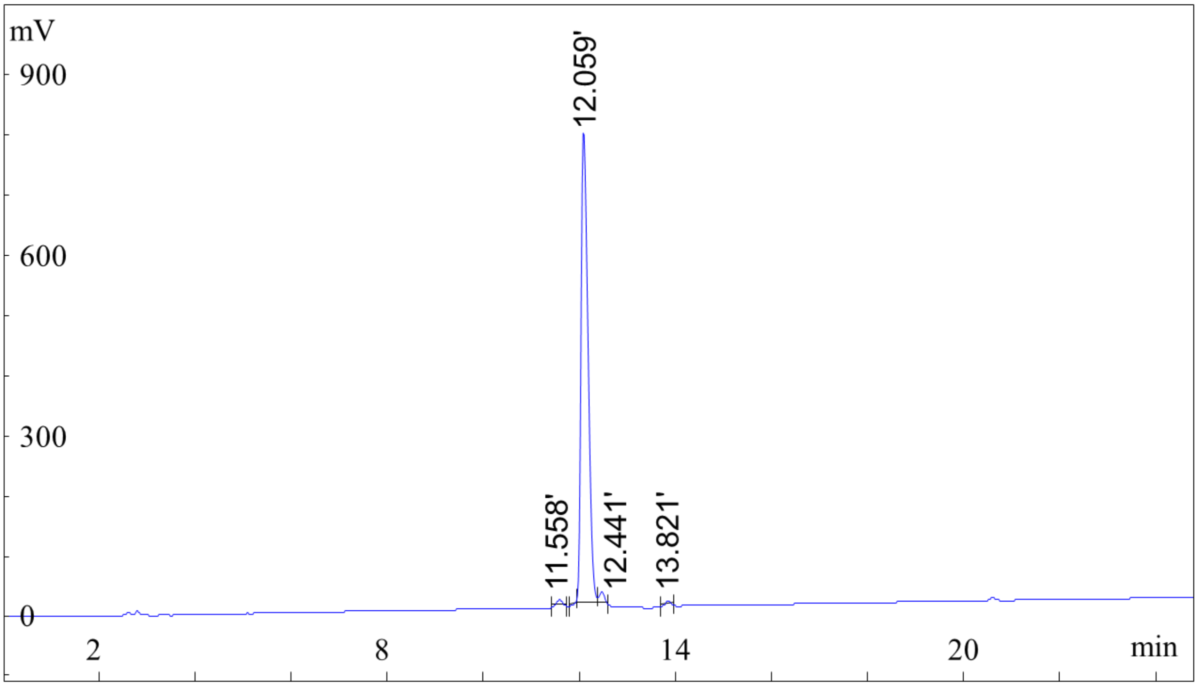

Figure S38. HPLC trace of **TI-7**.

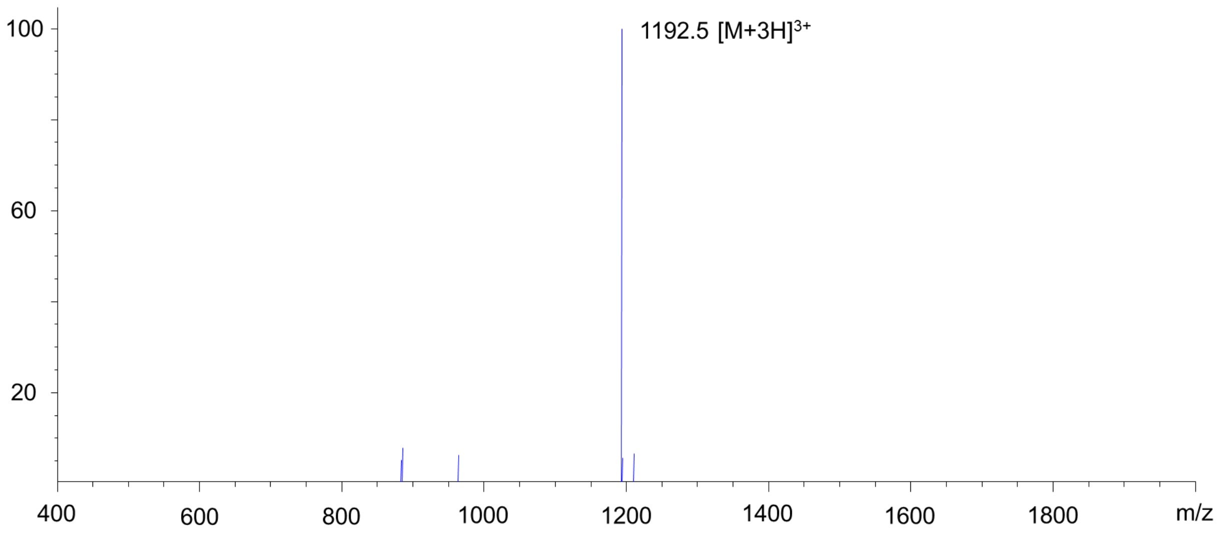

Figure S39. Mass spectrum of **TQ-26**.

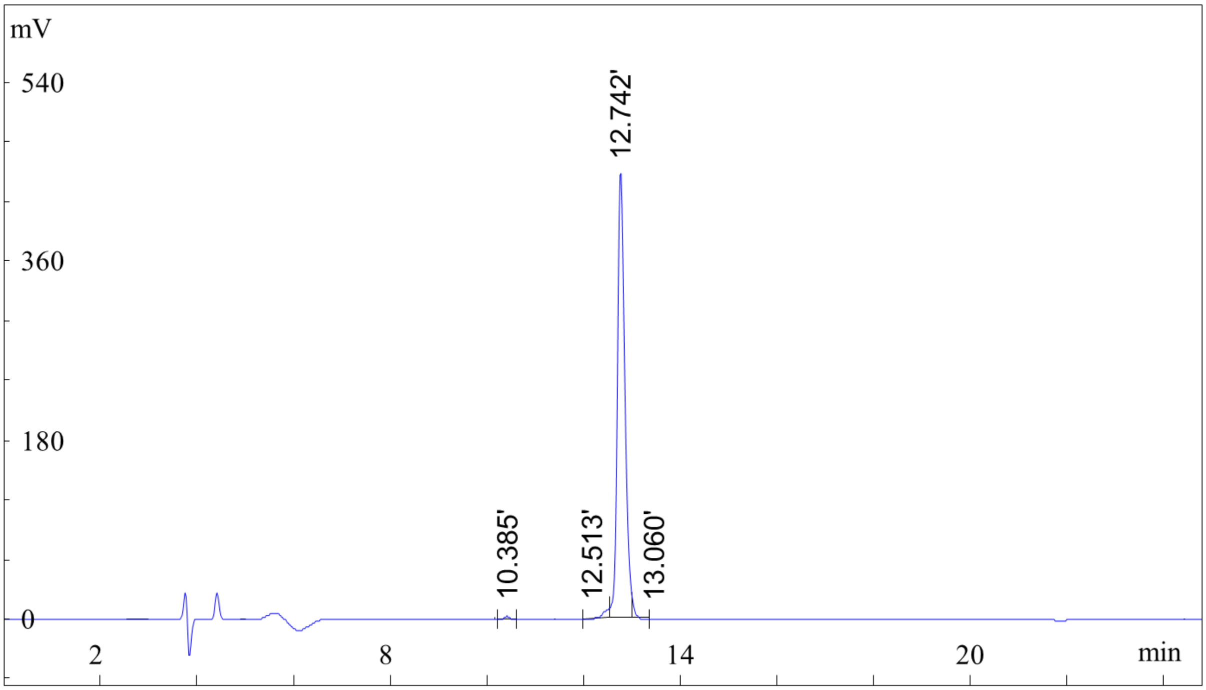

Figure S40. HPLC trace of **TQ-26**.

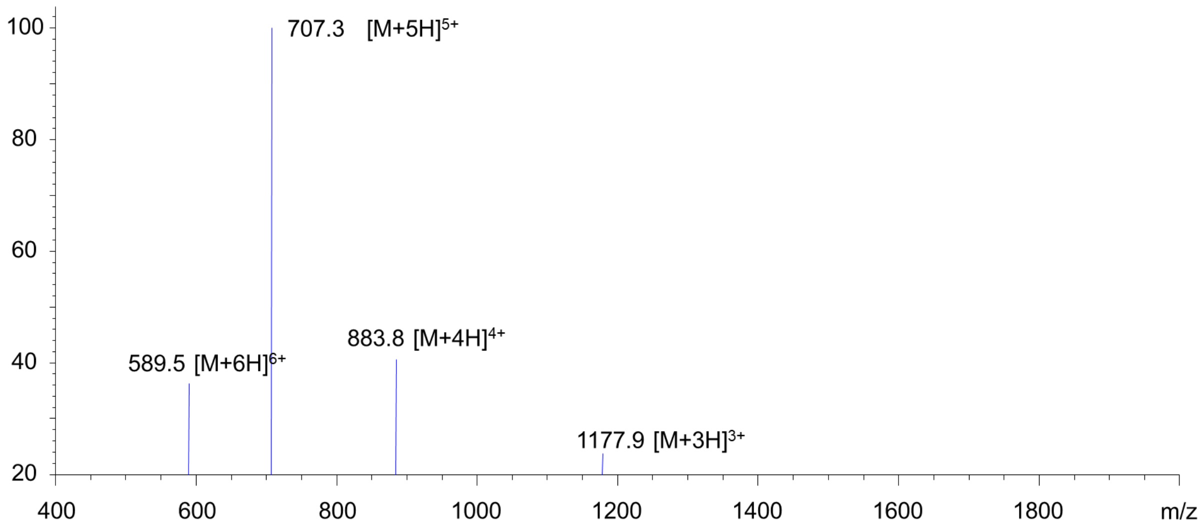

Figure S41. Mass spectrum of **TI-26**.

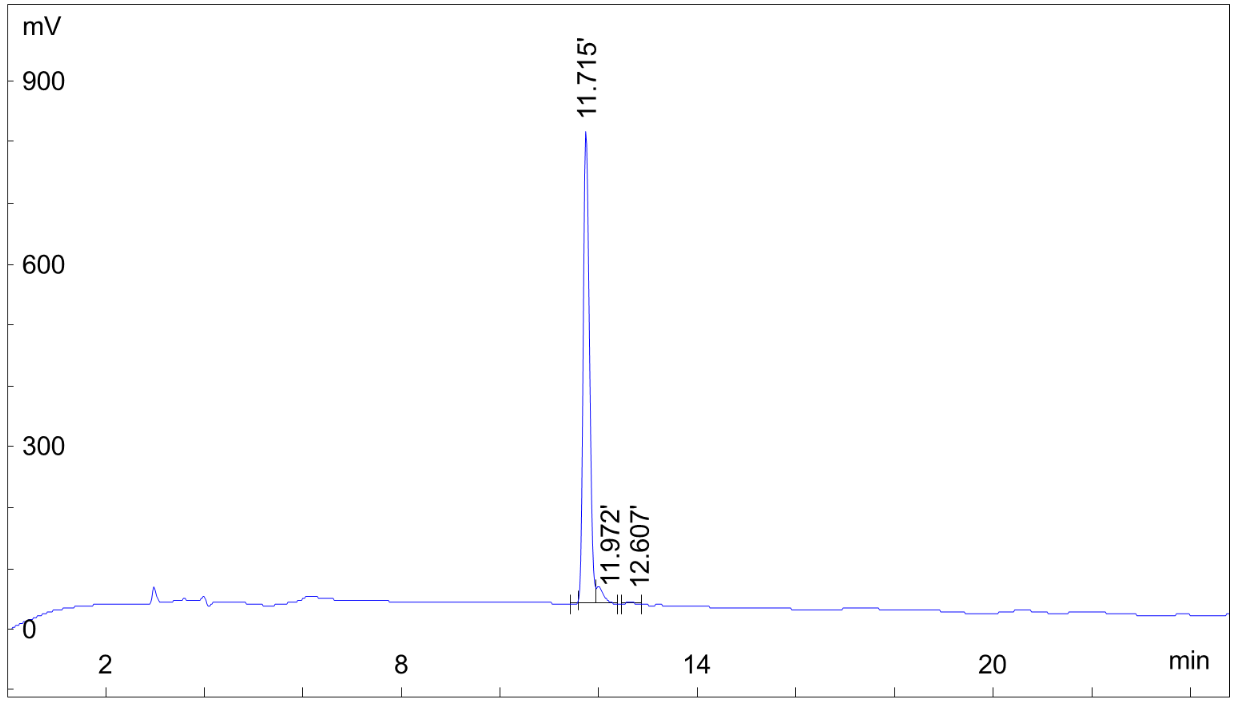

Figure S42. HPLC trace of **TI-26**.

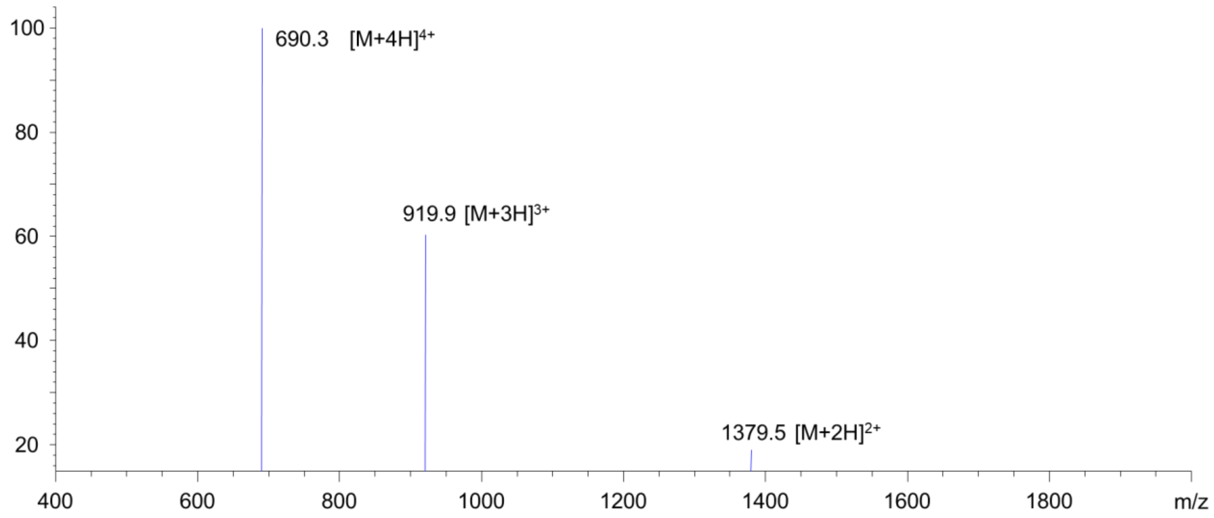

Figure S43. Mass spectrum of **TQ-19**.

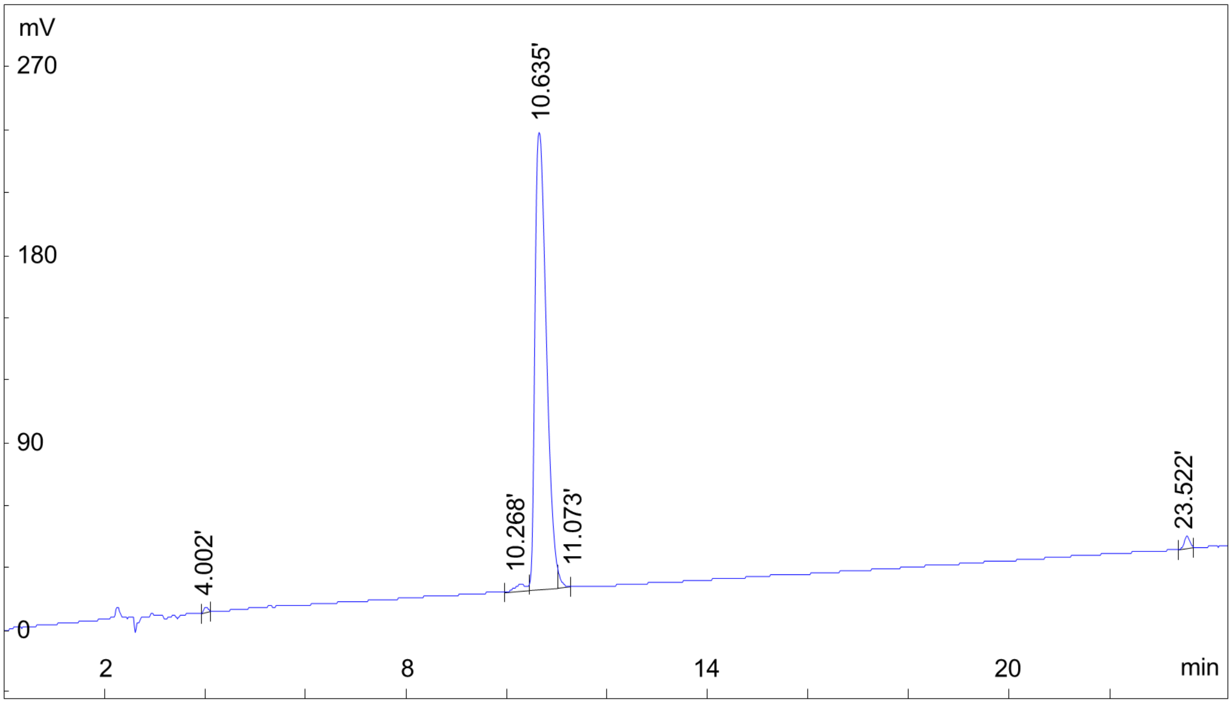

Figure S44. HPLC trace of **TQ-19**.

S3. Table

Table S1. Double reciprocal linear fitting equation determining binding constant (K_a) from fluorescence titration of peptide probes (2×10^{-6} M) in the presence of increasing CNTs (1–8 μg mL^{-1}), GO (1–15 μg mL^{-1}), 2D MnO_2 (1–12 μg mL^{-1}) and AuNPs (1–20 μg mL^{-1}) at the indicated concentrations.

Peptide/Material	K_a (μg mL^{-1})
**TL7**/CNTs	0.0776
**TL7**/GO	0.0319
**TL7**/2D MnO_2	0.0096
**TL7**/AuNPs	0.0249
**TI7**/CNTs	0.1462
**TI7**/GO	0.0273
**TI7**/2D MnO_2	0.1024
**TI7**/AuNPs	0.0153
**TQ19**/CNTs	0.1306
**TQ19**/GO	0.1574
**TQ19**/2D MnO_2	0.0980
**TQ19**/AuNPs	0.0425
**TQ26**/CNTs	0.0982
**TQ26**/GO	0.0613
**TQ26**/2D MnO_2	0.0607
**TQ26**/AuNPs	0.0488
**TI26**/CNTs	0.1570
**TI26**/GO	0.0815
**TI26**/2D MnO_2	0.1019
**TI26**/AuNPs	0.0602

Table S2. Naming rules of **S1-S20**

	GO	2D MnO_2	CNT	AuNPs
**TI7**	**S1**	**S2**	**S3**	**S4**
**TL7**	**S5**	**S6**	**S7**	**S8**
**TQ19**	**S9**	**S10**	**S11**	**S12**
**TI26**	**S13**	**S14**	**S15**	**S16**
**TQ26**	**S17**	**S18**	**S19**	**S20**

S4. Additional references

(1) Ma, Y. H.; Dou, W. T.; Pan, Y. F.; Dong, L. W.; Tan, Y. X.; He, X. P.; Tian, H.; Wang, H. Y. Fluorogenic 2D peptidosheet unravels CD47 as a potential biomarker for profiling hepatocellular carcinoma and cholangiocarcinoma tissues. *Adv. Mater.* **2017**, *29*, 1604253.

(2) Jumper, J.; Evans, R.; Pritzel, A.; Green, T.; Figurnov, M.; Ronneberger, O.; Tunyasuvunakool, K.; Bates, R.; Žídek, A.; Potapenko, A. Highly accurate protein structure prediction with AlphaFold. *Nature* **2021,** *596*, 583-589.

(3) Monteiro da Silva, G.; Cui, J. Y.; Dalgarno, D. C.; Lisi, G. P.; Rubenstein, B. M. High-throughput prediction of protein conformational distributions with subsampled AlphaFold2. *Nat. Commun.* **2024,** *15*, 2464.

(4) Pellegrini, L.; Albecka, A.; Mallery, D. L.; Kellner, M. J.; Paul, D.; Carter, A. P.; James, L. C.; Lancaster, M. A. SARS-CoV-2 Infects the Brain Choroid Plexus and Disrupts the Blood-CSF Barrier in Human Brain Organoids. *Cell stem cell* **2020,** *27*, 951-961 e5.

(5) Cantuti-Castelvetri, L.; Ojha, R.; Pedro, L. D.; Djannatian, M.; Franz, J.; Kuivanen, S.; van der Meer, F.; Kallio, K.; Kaya, T.; Anastasina, M.; Smura, T.; Levanov, L.; Szirovicza, L.; Tobi, A.; Kallio-Kokko, H.; Osterlund, P.; Joensuu, M.; Meunier, F. A.; Butcher, S. J.; Winkler, M. S.; Mollenhauer, B.; Helenius, A.; Gokce, O.; Teesalu, T.; Hepojoki, J.; Vapalahti, O.; Stadelmann, C.; Balistreri, G.; Simons, M. Neuropilin-1 facilitates SARS-CoV-2 cell entry and infectivity. *Science* **2020,** *370*, 856-860.

(6) Hashizume, M.; Takashima, A.; Ono, C.; Okamoto, T.; Iwasaki, M. Phenothiazines inhibit SARS-CoV-2 cell entry via a blockade of spike protein binding to neuropilin-1. *Antivir. Res.* **2023,** *209*.

(7) Garcia-Beltran, W. F.; St Denis, K. J.; Hoelzemer, A.; Lam, E. C.; Nitido, A. D.; Sheehan, M. L.; Berrios, C.; Ofoman, O.; Chang, C. C.; Hauser, B. M.; Feldman, J.; Roederer, A. L.; Gregory, D. J.; Poznansky, M. C.; Schmidt, A. G.; Iafrate, A. J.; Naranbhai, V.; Balazs, A. B. mRNA-based COVID-19 vaccine boosters induce neutralizing immunity against SARS-CoV-2 Omicron variant. *Cell* **2022,** *185*, 457-466 e4.

(8) Dou, W.-T.; Wang, X.; Liu, T.; Zhao, S.; Liu, J.-J.; Yan, Y.; Li, J.; Zhang, C.-Y.; Sedgwick, A. C.; Tian, H., A homogeneous high-throughput array for the detection and discrimination of influenza A viruses. *Chem* **2022**, *8*, 1750-1761.
